# Outcomes of Best-Practice Guided Digital Mental Health Interventions for Youth and Young Adults with Emerging Symptoms: Part I. A Systematic Review of Socioemotional Outcomes and Recommendations

**DOI:** 10.1007/s10567-024-00469-4

**Published:** 2024-03-15

**Authors:** Jessica E. Opie, An Vuong, Ellen T. Welsh, Richard Gray, Natalie Pearce, Sonia Marchionda, Rachel Mutch, Hanan Khalil

**Affiliations:** 1 School of Psychology & Public Health, The Bouverie Centre, La Trobe University, 8 Gardiner Street, Brunswick, Melbourne, VA 3056 Australia; 2 La Trobe University, Melbourne, VA 3000 Australia; 3 Latrobe University, Bendigo, VIC 3551 Australia; 4 Beyond Blue, Melbourne, VA 3000 Australia

**Keywords:** Systematic review, Youth, Adolescent, Young adult, Online, Mental health, Youth mental health, Digital

## Abstract

Youth-specific digital mental health interventions (DMHI) represent an emerging field of study, and aim to increase access, improve socioemotional outcomes, and, where required, support triage to targeted interventions. However, prior reviews have reported inconsistent findings on the clinical effectiveness of such interventions in young adults (12–25 years). Further, shortfalls remain for the impact of guided interventions based on the mode of delivery and the type of human support personnel (e.g., professional or peer) guiding the intervention. In response, this systematic review, co-designed with Australia’s leading mental health organization, aims to assess the effectiveness of guided digital programs in improving youth socioemotional outcomes. Included studies involve young people experiencing mental ill-health, receiving brief (i.e., 1–12 sessions), digitally delivered (at least partially) psychological interventions that were guided or partially guided, tested in a type of experimental study, with a socioemotional outcome. Specific socioemotional outcomes examined were depression, anxiety, stress, wellbeing, mindfulness, and quality of life. A systematic search of the contemporary published and grey literature identified 22,482 records with 32 relevant records published between 2018 and 2023. A narrative synthesis guided integration of findings. Results demonstrated strong evidence for the effectiveness of guided interventions on socioemotional outcomes (i.e., depression, anxiety, stress) yet these effects were short-lived. When factoring in the use of different control groups (i.e., active vs. inactive), inconsistent effects were observed for the socioemotional outcomes of depression, anxiety, and stress. The mode of delivery (i.e., asynchronous, synchronous, combined) and the type of human support personnel did not appear to impact socioemotional outcomes. Results indicate efficacious brief digital interventions for depression and anxiety include refresher/follow-up content, goal setting content, and relapse prevention content. In contrast, poor efficacy is associated with interventions that include homework tasks, self-monitoring, and log-keeping content.

*PROSPERO, ID* CRD42023405812.

**Supplementary Information:**

The online version contains supplementary material available at 10.1007/s10567-024-00469-4.

Youth mental illness represents an urgent public health concern requiring immediate action (Colizzi et al., 2020; Collishaw & Sellers, 2020). Globally, the World Health Organization (2020) estimates the aggregated global prevalence of youth and young adults (i.e., those aged 10–25 years) with a mental health disorder range from 10 to 20%. International rates of youth mental health symptoms and disorder have also sharply risen following the COVID-19 pandemic (Power et al., 2020; Stewart et al., 2023), and over recent decades (Keyes et al., 2019; Merikangas et al., 2009). Youth mental illness can result in immediate intrapersonal and interpersonal ramifications, and if unaddressed, can trigger a long-term cascading disability trajectory, resulting in costly personal, social, and economic outcomes (World Health Organization, 2021). Given the emerging and sub-diagnostic nature of many mental illness pathways, adolescence and emerging adulthood are opportune periods for preventative action. However, young adults are less likely to seek professional support for their mental health than those in older age groups (Babajide et al., 2020; Slade et al., 2009).

To effectively address these mental health concerns, there is a growing emphasis on youth-friendly, stigma-free, and accessible digital interventions (Hollis et al., 2017; Lehtimaki et al., 2021), such as digitally delivered mental health interventions. Digital mental health interventions (DMHIs) have emerged as vital resources, especially for young people in remote areas, those new to mental health services, and those seeking privacy and safety (Hollis et al., 2017; Lehtimaki et al., 2021; Pretorius et al., 2019; Schueller & Torous, 2020; Wilson, 2022). These DMHIs, encompassing online psychological interventions for individual or group therapy, and mobile services using calls, video meetings, or messaging, have evolved significantly since the 1980s (Burns et al., 2014; Marsac & Weiss, 2019; McNamee et al., 1989). Today’s platforms offer interactive, personalized content in both synchronous and asynchronous formats, aligning with the tech-savvy nature of today’s youth (Aschbrenner et al., 2019; Lattie et al., 2022; Philippe et al., 2022; Pokowitz et al., 2023).

In the present review, DMHIs refer to psychological interventions, for mental health conditions or symptoms, delivered online individually or to a group. They also include mobile phone services or applications involving voice calls, video meetings or text/chat messaging and can be live, automated, or pre-recorded.

## The Rise of Digital Mental Health Interventions

Initially, DMHIs were primarily designed to overcome the physical and economic barriers to accessing healthcare, while leveraging the ubiquitous nature of internet, mobile phone, and computer access. The COVID-19 pandemic accelerated the rapid expansion and uptake of these DMHIs mainly due to closures of typical in-person mental health providers (Mahoney et al., 2021). The pandemic resulted in increases in the incidence of mental ill-health, further increasing demand for telemedicine services, with attendant increased burden on the healthcare system and demand for DMHIs (McLean et al., 2021). The confluence of these factors has resulted in a substantial increase in the development, uptake, and research of DMHIs during the COVID-19 pandemic and beyond (Celia et al., 2022; Cerutti et al., 2022).

The clinical efficacy of DMHIs is promising, revealing many of these interventions to be equivalent to their in-person counterparts (Andrews et al., 2018). Research examining digital mental health platforms suggests the promise for improved service accessibility and engagement, with more people being treated at a lower cost (Lattie et al., 2022; Sherifali et al., 2018). However, while the evidence-base for the clinical benefits of DMHIs is strong for adults, it currently represents an emerging field of research for youth-specific DMHIs, with calls for greater research enquiry (Lattie et al., 2022). These DMHIs are especially well-suited to young people who tend to be technologically savvy and early adopters of such approaches (Aschbrenner et al., 2019; Giovanelli et al., 2020). DMHIs have also been found to be particularly well suited for people who are deemed (or seen) to be at ‘less risk’ (i.e., not in an acute psychiatric emergency and without currently meeting clinical diagnostic thresholds) (Paganini et al., 2018; Rigabert et al., 2020), which includes universal, selective, and indicated prevention. Given the promise that these digital interventions hold, it is unsurprising that digital mental health is now a burgeoning field of study. DMHIs could be particularly useful for people who face stigma accessing mental health services or for youth who are reluctant to ask parents for consent accessing these services (Lattie et al., 2022).

## Obstacles to Optimized Digital Health Services

Despite the recent rapid growth and identified benefits of self-guided (i.e., 100% self-guided digital delivery) DMHIs, concerns regarding their sustained usage, appropriate utilization, and ongoing efficacy have been raised (Mehrotra et al., 2017; Opie et al., 2023; Schueller et al., 2017). Self-guided DMHIs appear to have high attrition rates, limiting the impact of such interventions (Alqahtani & Orji, 2019; Karyotaki et al., 2015). Furthermore, there is currently a limited understanding of the factors contributing to such intervention attrition and specifically understanding how these retention rates can be improved (Alqahtani & Orji, 2019; Schmidt et al., 2019). Ethical concerns pertaining to these DMHIs are also important to consider, including the storage and sharing of personal data and risk management associated with distant, independent access (Galvin & DeMuro, 2020; Wykes et al., 2019). Additionally, person-specific influences can impact the usage (or lack thereof) of intervention design, such as motivation and capability, which are currently under researched (Cross et al., 2022). These influences may include low digital literacy, negative prior user experience, or costs associated with internet or program access. These limitations may prevent users from reaping the full benefits of these interventions (Schueller et al., 2017).

## DMHIs with a Guided Component

To address these problems, researchers have turned to DMHIs with guided support. DMHIs with guided support includes human contact embedded within their DMHI delivery. Such guided support aims to to enhance socioemotional outcomes, engagement, and to provide clinical and technical support (Heber et al., 2017; Werntz et al., 2023). Methods of DMHIs can be partially guided (i.e., combination of guided and self-guided intervention elements) or completely guided (i.e., 100% delivered by human support). Such support can be delivered synchronously (i.e., live support occurring in real-time; e.g., videoconferencing, phone call) and/or asynchronously (i.e., delayed; e.g., email, text message), by an array of human support providers, including qualified mental health clinicians (e.g., psychologists) and non-clinician or paraprofessional support (e.g., lived experience peer support workers, lay counselors, volunteers, or students). Of note, heterogeneity in these guided supports is evident varying in terms of support content, amount, and timing, for example, which may introduce measurement error when attempting to compare these interventions (Harrer et al., 2019).

## Existing Systematic and Meta-analytic Reviews

While not youth-specific, prior meta-analytic evidence demonstrates the efficacy of DMHIs with partially and/or fully guided support for depression (Karyotaki et al., 2021), anxiety (Olthuis et al., 2016a), and post-traumatic stress disorder (Olthuis et al., 2016b). Further, meta-analytic evidence has shown such DMHIs with human support to be equivalent to their face-to-face counterparts (Andrews et al., 2018; Cuijpers et al., 2019). One meta-analysis examined the efficacy of DMHIs with non-clinical support to self-guided, and clinician-guided DMHIs (Leung et al., 2022). Notably, they reported no significant difference between clinician-guided and non-clinician-guided DMHIs in terms of intervention efficacy. They also found a significant difference in effectiveness between self-guided and non-clinician-guided DMHIs, favoring non-clinical guided support. They found non-clinician-guided DMHIs reported significantly greater post-treatment efficacy relative to controls. However, results were based on studies which included participants aged 16–64, and thus was not youth specific.

### Youth Populations

When looking at youth populations, meta-analytic and systematic review evidence remains mixed. Meta-analytic evidence has reported varying effect sizes (Hedges’ *g* range 0.46 to 0.94; Cohen’s *d* range 0.14 to 0.33) when comparing DMHIs against a control condition (Bennett et al., 2019; Ebert et al., 2015; Garrido et al., 2019; Ma et al., 2021). Systematic reviews have also examined the efficacy of guided, partially guided, and unguided youth-specific DMHIs, with findings indicating overall improvements in depression, stress, and anxiety outcomes (Hollis et al., 2017; Lehtimaki et al., 2021; Zhou et al., 2021); however inconsistent effects have been identified when factoring in different control conditions (e.g., active control (receives an alternative intervention concurrent to intervention group) versus inactive control (receives no intervention above treatment as usual) (Hollis et al., 2017; Lehtimaki et al., 2021; Zhou et al., 2021). Additionally, such differences have been attributed to within-study or within-intervention heterogeneity in terms of sampling, delivery, and content (Lehtimaki et al., 2021; Zhou et al., 2021).

### Indicated Youth Populations

The scope of youth populations in DMHI research varies. Notably, van Doorn et al. (2021) uniquely concentrated on indicated preventive interventions for youth exhibiting emerging symptoms, unlike other reviews that merged both universal and indicated prevention population (Ebert et al., 2015; Harrer et al., 2019). This approach by van Doorn et al. highlighted that DMHIs have a more pronounced effect on indicated youth with emerging symptoms compared to universal youth without symptoms (Conley et al., 2016).

Given the mixed and emerging findings from various systematic and meta-analytic reviews of youth DMHI efficacy, it is unsurprising that there have been calls for further research into the efficacy of DMHI guided human supports based on these mixed and emerging findings (Bennett et al., 2019; Ebert et al., 2015; Garrido et al., 2019).

## The Need for Further Systematic Examination

Considering the limitations and advantages of such DMHIs, their rapid growth warrants further systematic examination to build upon the existing literature that has supported their efficacy. While DMHIs appear to work better than no intervention to improve depression in young people, they may only be of clinical significance when use is highly supervised (Garrido et al., 2019). The ability of DMHIs to deliver automated and self-directed interventions is frequently argued as a way to improve access to mental health services and avoid stigma; however, inconsistencies in intervention efficacy have been reported (Baumeister et al., 2014; Dear et al., 2016; Hollis et al., 2015; Josephine et al., 2017).

While there is a plethora of research on the benefits and disadvantages on fully self-guided interventions as described above, further research is needed to understand the efficacy of different types of guided DMHIs, including synchronous and asynchronous delivery methods, and their comparative variations in efficacy of programs delivered via various channels (Rogers et al., 2021). Attention to socioemotional data is also needed to provide an efficacious and impactful intervention for young people (Garrido et al., 2019; Lehtimaki et al., 2021; Rogers et al., 2021). Taken together, existing systematic reviews have highlighted the importance of guided support in DMHIs for young people.

Previous systematic reviews (Baumeister et al., 2014; Harrer et al., 2019) have also not fully explored the specific elements and characteristics that contribute to the efficacy of DMHIs. Recognizing and understanding these key characteristics is essential for guiding future research. This insight is crucial for enhancing the effectiveness of current digital tools and employing the latest technologies more effectively to support this vulnerable population. Understanding these aspects can lead to significant improvements in how digital mental health resources are developed and utilized.

As a research priority, is a recognized need for more systematic research into the impact of human-guided DMHIs. This includes examining the impact of various types of support personnel, including clinicians, trained laypersons, and peers with lived experience, as well as examining the different levels of guidance they provide, from partially to fully guided support (Hollis et al., 2017; Ma et al., 2021). Additionally, research gaps remain in understanding the effects of synchronous and asynchronous DMHIs on clinical effectiveness and treatment adherence (Hollis et al., 2017). Addressing these gaps and limitations of previous systematic reviews is essential for development of effective and accessible mental health care.

## The Current Study

To address the limitations identified in existing systematic reviews, as detailed above, the current review expands upon the literature by evaluating the body of research on youth-specific DMHIs that offer some level of guidance. Our approach includes identifying and synthesizing all youth-focused DMHIs that are either fully or partially guided by human support. The objective is to comprehensively report on the socioemotional clinical efficacy outcomes of these guided and partially guided youth DMHIs.

## Methods

A systematic review methodology utilized the Joanna Briggs Institute (JBI) methodology framework (Aromataris & Munn, 2020). Our reporting adhered to the Preferred Reporting Items for Systematic Reviews and Meta-Analyses (PRISMA; Page et al., 2021). See Online Resources 1 for a complete PRISMA checklist. A protocol of the present review was prospectively registered in PROSPERO (March 23, 2023; CRD42023405812).

The review methodology was co-designed and conducted alongside Beyond Blue, Australia’s most well-known and visited mental health organization. This review was also conducted by several lived experience consumer academics. Thus, this review was informed by consumer principles, acknowledging the meaningful contributions that people with a lived experience have to offer whose experiences and perspectives are to be respected and valued. Collectively, the current review aimed to bring together academic, consumer, and mental health service skills, experiences, and voices.

### Inclusion Criteria

The Population, Intervention, Comparator, Outcome, and Study design (PICOS) framework (McKenzie et al., 2019) guided inclusion criteria eligibility (See Table 1). If necessary information was not reported in-text, the study was excluded. Only literature written in English language was included.

**Table 1:** PICOS framework

Concept	Concept details
Population (P)	Youth (12–25 years, inclusive) experiencing non-acute, emerging, mild-to-moderate mental ill-health symptoms. Therefore, we examined indicated populations and excluded universal and selected prevention populations, as well as all treatment and recovery populations. Studies were also excluded if participants had an existing psychiatric diagnosis. We also included studies whose sample fell outside of 12–25, provided the study’s mean age was within 12–25 years (inclusive)
Intervention (I)	Interventions were youth and young-adult specific, intended for those aged between 12 and 25 years. General adult interventions were excluded. The scope of interventions was mental health. Combination interventions that focus on mental ill-health *and* alcohol and other drugs (AOD) interventions were also included. Entirely AOD interventions were excluded. Interventions were evidence-based or informed and developed by a mental health expert (clinician, researcher, and/or expert by experience). The intervention duration was brief, defined as intervention length ranging from 1 to 12 sessions and duration ranging from 0 to 12 months. Interventions were standardized and manualized (solely or partially). The intervention was digitally delivered by any digital delivery method (e.g., telehealth, email texts, online chats smartphone applications). Interventions were individually delivered, with dyadic or group-based interventions excluded. Intervention delivery channel could be: 1. Combination delivery (partially guided *and* partially self-guided) or 2. Entirely guided. Such guided delivery could be synchronous (i.e., live contact) or asynchronous (delayed contact). Guidance could include support from a clinician, researcher, expert by experience, or a mix of experts. Self-guided interventions were excluded. There were no theoretical framework parameters around included interventions
Comparison (C)	To be included in this review, studies contained between group data with comparison group being any of the following: placebo, control, group receiving an equivalent in-person program, or any other varied intervention. Thus, no comparisons were imposed. Within-group studies were also included (i.e., where no comparison group data were included)
Outcome (O)	All studies were required to report on pre-post intervention socioemotional outcomes
Study design (S)	Primary research from published and unpublished sources in the form of experimental and quasi-experimental (i.e., randomized controlled trials, non-randomized controlled trials, before and after studies, and interrupted time-series studies) were included. Case control studies were also included. All included studies needed to report on clinical pre-post mental health program efficacy data related to reducing psychological distress

### Types of Sources

The search was limited to contemporary published literature. Full text references in English were searched from 14 March 2018- 14 February 2023. Date restrictions were applied to the search to ensure that we conducted a contemporary examination of the literature due to rapid recent technological advancements and associated technological redundancies. Date restrictions were also applied due to the dearth of available literature pre-2018. This decision was further made to allow for evaluations of comparable digital youth-specific interventions.

### Search Strategy

We followed a four-step search strategy. An initial limited search of PsycINFO was conducted, followed by analysis of the text contained in the title and abstract, and of the index terms used to describe the article. This identified the keywords and index terms used for a second search across all the databases covered by this study. The second search was a systematic search of five electronic databases: PsycINFO (Ovid), MEDLINE (Ovid), CINAHL (EBSCO), Cochrane Central Register of Controlled Trials (Central; via Cochrane Library). See Online Resources 2 for a complete search strategy (concept and terms) of all included databases. The third search was an examination of unpublished and grey literature. This included identifying dissertations and theses identified via ProQuest Dissertations and Theses. Global Trial registries were also searched to identify ongoing studies or complete but unpublished studies, these included Australian New Zealand Clinical Trial Register (https://www.anzctr.org.au/) and ClinicalTrials.gov. The first 20 pages of Google were also searched. See Online Resources 3 for a complete grey literature search strategy. Finally, to ensure a comprehensive search was conducted, reference lists of all eligible studies and pertinent systematic reviews were manually searched to identify further studies that met inclusion criteria. Authors were not contacted for missing data.

#### Study Screening and Selection

All records were imported to Endnote (2013) where duplicates were removed. Remaining studies were imported in Covidence (Veritas Health Innovation, 2020) and were screened at title and abstract level by four reviewers (JO, AV, SM, EW). Studies were then screened at full-text level. At both title and abstract, and full-text, 75% of records were double screened.

#### Data Extraction

Data extraction was completed by four independent reviewers (JO, AV, SM, EW) with disagreements resolved through conferencing. Data from each full-text article were charted by one reviewer and checked by a second independent reviewer. Data were extracted into a priori standardized data extraction forms, consistent with Tables 3, 4 and 5.

### Quality Assessment

To appraise methodological quality of included papers, we ranked studies based upon study design. Upon appraisal completion, studies were labelled as ‘weak’, ‘moderate’, or ‘high’ in terms of their methodological quality. An a priori decision was made not to exclude any record based on study quality. All studies were appraised via the Quality Assessment Tool for Quantitative Studies (EPHPP, 2010). Quality appraisal checklist response options were ‘yes’, ‘no’, ‘unclear’, or ‘not applicable’. Grey literature was critically assessed using the Authority, Accuracy, Coverage, Objectivity, Date, and Significance (AACODS) checklist (Tyndall, 2010). Studies were subsequently grouped into low risk (> 75% of quality criteria met), moderate risk (> 50% of criteria met), or high risk of bias (< 50% criteria met). An a priori decision was made not to exclude studies based on quality. One author assessed study quality for all the papers, and a second author independently assessed the study quality of 25% of the papers (IRR = 75% agreement). All disagreements were resolved through conferencing.

### Synthesis

Included studies were categorised under sub-headings, consistent with Tables 2, 3, 4. To identify socioemotional outcome efficacy and user experience outcomes, we collated and categorized the extracted intervention characteristics and outcomes. Outcomes of examination were data-driven, wherein we privileged frequently reported outcomes. Due to data heterogeneity, a meta-analysis was not feasible, and results were narratively synthesized. If two included studies reported on an identical outcome, only data from the study with the largest sample size was included for that outcome. Where a dissertation and a published record reported on an identical study, the published paper was included and the dissertation excluded, as the published paper had passed the peer-review process.

**Table 2:** Study quality of included studies

Published papers—EPHPP quality assessment tool for quantitative studies							
Author (Year)	A. Selection BIAS	B. Study design	C. Confounders	D. Blinding	E. Data collection methods	F. Withdrawals & drop-outs	Global rating*
Celia et al. (2022)	Moderate	Moderate	Moderate	Moderate	Strong	Strong	Strong
Cerutti et al. (2022)	Strong	Moderate	Strong	Moderate	Strong	Strong	Strong
Cook et al. (2019)	Moderate	Strong	Weak	Moderate	Strong	Moderate	Moderate
Grudin et al. (2022)	Moderate	Strong	Weak	Moderate	Strong	Strong	Moderate
Garnefski and Kraaij (2023)	Moderate	Moderate	Weak	Moderate	Moderate	Moderate	Moderate
Harra and Vargas (2023)	Moderate	Strong	Strong	Moderate	Strong	Moderate	Strong
Hennemann et al. (2022a)	Strong	Strong	Moderate	Moderate	Moderate	Strong	Strong
Hennemann et al. (2022b)	Strong	Strong	Strong	Moderate	Moderate	Strong	Strong
Juniar et al. (2022)	Strong	Moderate	Weak	Moderate	Strong	Weak	Moderate
Karyotaki et al. (2022)	Strong	Strong	Weak	Moderate	Strong	Strong	Moderate
Keinonen et al. (2021)	Strong	Moderate	Moderate	Moderate	Strong	Strong	Strong
Klimczak et al. (2023)	Moderate	Strong	Weak	Moderate	Strong	Moderate	Moderate
Küchler et al. (2023)	Strong	Strong	Moderate	Moderate	Strong	Weak	Strong
Lappalainen et al. (2021)	Weak	Strong	Weak	Moderate	Strong	Strong	Weak
Lappalainen et al. (2023)	Moderate	Strong	Weak	Moderate	Strong	Weak	Moderate
Novella et al. (2022)	Strong	Strong	Weak	Moderate	Strong	Strong	Moderate
O'Connor et al. (2020)	Strong	Strong	Moderate	Moderate	Strong	Moderate	Strong
O'Connor et al. (2022)	Moderate	Strong	Weak	Moderate	Strong	Weak	Weak
Pescatello et al. (2021)	Moderate	Moderate	Strong	Weak	Strong	Weak	Weak
Peynenburg et al. (2022)	Moderate	Strong	Weak	Moderate	Strong	Moderate	Moderate
Radomski et al. (2020)	Moderate	Strong	Weak	Moderate	Moderate	Weak	Weak
Radovic et al. (2021)	Moderate	Strong	Weak	Moderate	Strong	Moderate	Moderate
Ravaccia et al. (2022)	Moderate	Moderate	Weak	Moderate	Strong	Weak	Weak
Rice et al. (2020)	Moderate	Moderate	Weak	Moderate	Strong	Strong	Moderate
Rodriguez et al. (2021)	Strong	Strong	Weak	Moderate	Strong	Weak	Moderate
Schueller et al. (2019)	Strong	Moderate	Weak	Moderate	Moderate	Strong	Moderate
Sit et al. (2022)	Moderate	Moderate	Weak	Moderate	Moderate	Weak	Weak
Stapinski et al. (2021)	Strong	Strong	Moderate	Moderate	Strong	Moderate	Strong
Sun et al. (2022)	Moderate	Strong	Weak	Moderate	Strong	Strong	Moderate
van Doorn et al. (2022)	Moderate	Moderate	Weak	Moderate	Moderate	Strong	Moderate

Unpublished papers—AACODS Checklist							
	Authority	Accuracy	Coverage	Objectivity	Date	Significance	%
Koltz (2022)	Yes	Yes	Yes	Yes	Yes	Yes	100 = Low risk of bias
Wahlund (2022)	Yes	No	Yes	Yes	Yes	Yes	83.33 = Low risk of bias

Published *Criteria for global rating; 1. **Strong** = no weak ratings; 2. **Moderate** = one weak rating; 3. **Weak** = two or more weak ratings. Unpublished: Criteria for risk of bias: (1) low risk of bias (75% of quality criteria met); (2) moderate risk of bias (> 50% of quality criteria met, and (3) high risk of bias (< 50% quality criteria met)

**Table 3:** Characteristics of included studies

Total sample							
Study (Year) Country, *Recruitment*	Design (# arms)	Mental health concern	*M* age (range) F%	Pre *N* (AR%)	Name		*n* (AR%)
App-based (accessed through smartphone/tablet)							
Ravaccia et al. (2022) UK, *School*	Mixed-method (pre-post) (1-arm)	General well-being	NR 64%F	398 (80)	Tellmi		398 (80)
Schueller et al. (2019) USA, *Community*	Pilot feasibility trial (pre-post) (1-arm)	Mental wellbeing	19.06 (18–24) 65F%	28 (18)	Pocket helper + Purple chill + Slumber time		28 (18)
Sit et al. (2022) China, *University*	Exp. (pre-post) (1-arm)	Depression Anxiety	NR (18–25) 68F%	38 (66)	Step-by-step (SbS)		38 (66)
Sun et al. (2022) China, *Community*	RCT (2-arm)	Anxiety Depression	22.21 (> 18) 73.7F%	114 (13)	WeChat mini		Mindfulness-mHealth: 57 (9) Social support- mHealth: 57 (18)
Combination delivery (e.g., Telehealth and app-based)							
Garnefski and Kraaij (2023) Netherlands, *Community*	Exp. (pre-post) (1-arm)	Depression	24.71 (> 18) 77F%	31 (26)	Moodpep		31 (26)
Hennemann et al. (2022a) Germany,*University*	RCT (2-arm)	Somatic symptom distress	24.53 (≥ 18) 83F%	156 (13)	iSOMA-guided		81 (16)
Hennemann et al. (2022b) Germany, *University*	RCT (2-arm)	Somatic symptom distress	24.60 (≥ 18) 83F%	149 (6)	iSOMA-guided		iSOMA-guided: 81(0) iSOMA GoD: 68 (0)
Klimczak et al. (2023) USA, *University*	RCT (3-arm)	Depression Anxiety	22.53 (≥ 18) 75F%	230 (24)	ACT guide		Phone: 77 (22) Text: 75 (21)
Lappalainen et al. (2021) Finland, *School*	RCT (3-arm)	Depression Psychological flexibility	15.27 (14–16) 51F%	243 (2)	Youth COMPASS		iACT-WhatsApp: 80 (0)
Lappalainen et al. (2023) Finland, *School*	RCT (3-arm)	Depression Psychological flexibility	15.01 (14–16) 67F%	234 (41)	Youth COMPASS		Student & virtual coach: 79 (48) Virtual coach only: 75 (56)
O'Connor et al. (2020) Canada, *Community*	Pilot RCT (2-arm)	Anxiety	15.3 (13–17) 90F%	94 (26)	Being real, easing anxiety: Tools helping electronically (BREATHE)		36 (0)
O'Connor et al. (2022) Canada, *Community*	RCT (2-arm)	Anxiety	16.4 (NR) 72F%	563 (57)	Being real, easing anxiety: Tools helping electronically (BREATHE)		258 (66)
Radomski et al. (2020) Canada, *Community*	RCT (2-arm)	Anxiety	16.6 (13–17) 71F%	536 (57)	Being real, easing anxiety: Tools helping electronically (BREATHE)		258 (67)
Rodriguez et al. (2021) China, *University*	RCT (2-arm)	Depression, Anxiety, Stress	23.5 (NR) 74F%	54 (57)	MIND		27 (41)
Stapinski et al. (2021) Australia, *Community*	RCT (2-arm)	Anxiety, Alcohol use	21.6 (17–24) 67F%	123 (28)	Inroads		62 (0)
van Doorn et al. (2022) Netherlands, *Community*	Exp. (pre-post) (2-arm)	Perceived stress	22.38 (NR) 100F%	8 (0)	ENYOY, Sense-IT		8 (0)
Telehealth (Zoom/videoconferencing software)							
Harra and Vargas (2023) USA, *University*	RCT (2-arm)	Anxiety, Depression	19.5 (NR) 47F%	45 (29)	Unnamed		14(33)
Novella et al. (2022) USA, *University*	RCT (2-arm)	Anxiety	19.29 (18–22) 87%F	52 (5)	Unnamed		23 (0)
Web-based (accessed through internet browser or internet supported device. e.g., computer, phone)							
Celia et al. (2022) Italy, *University*	Exp. (pre-post) (1-arm)	Stress, Anxiety, Social maladjustment, Negative affect	22.88 (NR) 65.6F%	32 (0)	Unnamed		32 (0)
Cerutti et al. (2022) Italy, *University*	Exp. (pre-post) (1-arm)	Depression, Anxiety, Hopelessness, Burnout	23.27 (NR) 78F%	67 (0)	Unnamed		67 (0)
Cook et al. (2019) UK, *University*	RCT (3-arm)	Worry/rumination	NR (18–24) 83F%	235 (31)	RESPOND		82 (39)
Grudin et al. (2022) Sweden, *Mental health service*	RCT (3-arm)	Experiential avoidance, Depression	15.4 (13–17) 59F%	32 (0)	Internet behavioral activation (I-BA)		11 (9)
Juniar et al. (2022) Indonesia, *University*	Feasibility study (pre-post) (1-arm)	Stress	24.03 (19–42) 85%	68 (63)	Rileks		68 (63)
Karyotaki et al. (2022) Netherlands, *University*	RCT (2-arm)	Depression, Anxiety	21.91 (≥ 18) 81F%	100 (18)	ICare Prevent		48 (17)
Keinonen et al. (2021) Finland, *University*	Exp. (1-arm)	Avoidance Depression	15 (14–16) NR	123 (0)	Unnamed		123 (0)
Koltz (2022) USA, *School*	Single case design (pre-post) (1-arm)	Stress	13.5 (12–15) 50F%	4 (0)	inSPIRE		4 (0)
Küchler et al. (2023) Germany, *University*	RCT (3-arm)	Mental wellbeing	25.77 (> 18 yr) 75F%	386 (48)	StudiCare-M		130 (58)
Pescatello et al. (2021) USA, *University*	Exp. (3-arm)	Psychological distress	NR (≥ 18 yr) NR F%	5568 (NR)	SilverCloud (SC)		SC:1,247 (NR) SC + therapy: 527 (NR)
Peynenburg et al. (2022) Canada, *University*	Randomized Factorial Trial (4-arm)	Depression, Anxiety	23.73 (17–46) 81F%	277 (30)	UniWellbeing		MI + Booster: 68 (29)
Radovic et al. (2021) USA, *Mental health service*	RCT (2-arm)	Depression, Anxiety	16 (12–19) 76F%	38 (34)	Supporting our valued adolescent (SOVA)		18 (22)
Rice et al. (2020) Australia, *Mental health service*	Exp. (pre-post) (1-arm)	Social anxiety	19.8 (14–25) 47F%	89 (15)	Entourage		89 (15)
Wahlund^ (2022) Sweden, NR	Pilot (pre-post) (1-arm)	Excessive worry	NR (13–17) NR F%	13 (8)	BIP Worry		13 (8)

^ = Unpublished thesis, *ACT* acceptance commitment therapy, *App* application, *AR* attrition rate, *E* electronic, *Exp* experimental, *F* female, *GoD* guidance on demand, *iACT* Internet-based ACT, *i-BA* Internet-based Behavioral Activation, *Incl* includes/including, *M* mean, *mHealth* mobile health, *MI* motivational interviewing, *N* sample size, *n* subsample size, *NR* not reported, *RCT* randomized controlled trial, *Yr* year

**Table 4:**
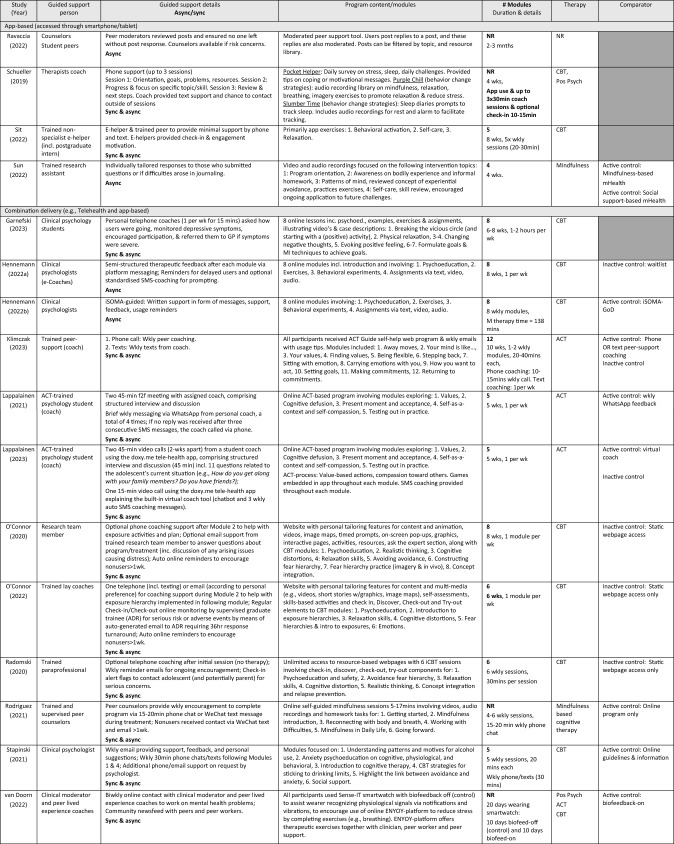

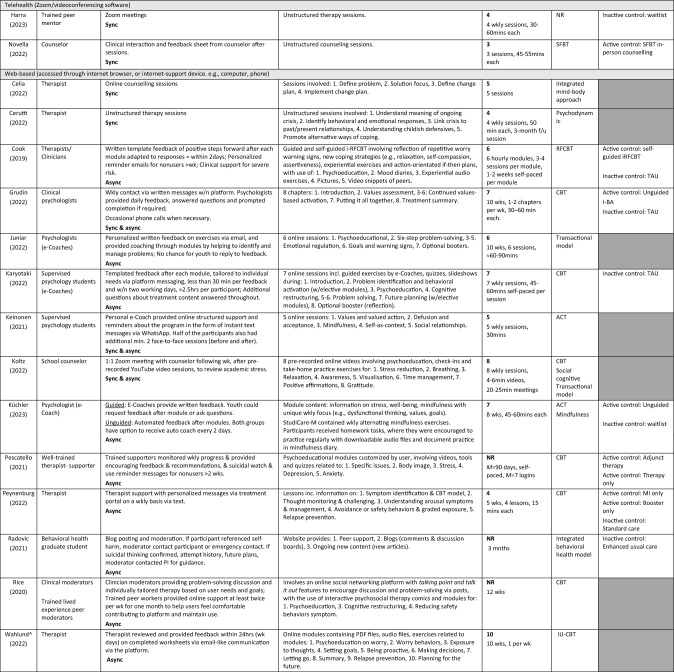
Characteristics of interventions

^^^ = Unpublished thesis; 1:1 = one-to-one, *ACT* acceptance commitment therapy, *Active control* Alternative intervention received, *ADR* adverse detection reviewer, *App* application, *Async* asynchronous, *Auto* automated, *Biofeed* biofeedback, *Biwkly* biweekly, *CBT* cognitive behavioral therapy, *Cog* cognitive, *E* electronic, *f2f* face-to-face, *GoD* guidance on demand, *iACT* internet-based ACT, *i-BA* internet-based behavioral activation, *iCBT* internet-based CBT, *Inactive control* no intervention received, *Incl* includes/including, *iRFCBT* internet-based RFCBT, *IU* intolerance of uncertainty, *M* mean, *Min/s* minute/s, *mHealth* mobile health, *MI* motivational interviewing, *NR* not reported, *Pos Psych* positive psychology, *RFCBT* rumination-focused CBT, *SFBT* solution-focused brief therapy, *Sync* synchronous, *TAU* treatment as usual, *Wk* week, *Wkly* weekly, *W/* with, *W/n* within, Grey shading = comparator not included in study; Dark gray shading = No comparator group

### Outcomes

Socioemotional outcomes examined were depression, anxiety, stress, wellbeing, mindfulness, and quality of life.

## Results

### Study selection

**Fig. 1:**
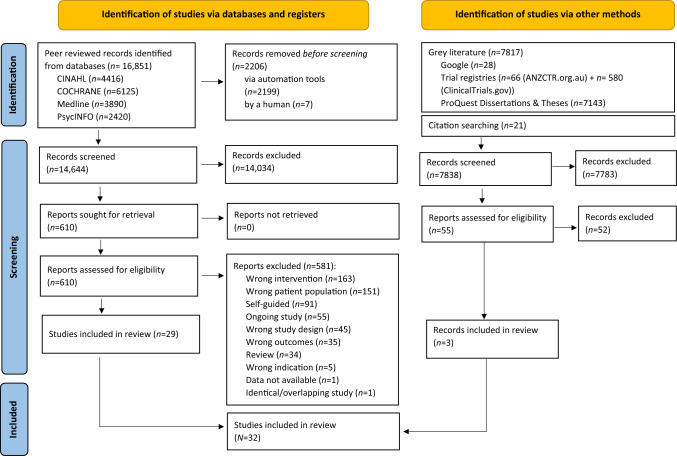
PRISMA diagram of the phases of the review process and record selection

The systematic literature search yielded 22,482 records (after removal of duplicates), of which 22,450 records were excluded at title/abstract (*n* = 21,817) and full-text level (*n* = 633). Double screening at title and abstract resulted in inter-rater reliability (IRR) of published literature 96% (*κ* = 0.43) and unpublished literature IRR 98% (*κ* = 0.45). At full-text, double-screening IRR was 98% (*κ* = 0.74) for published literature and 92.31% (*κ* = 0.75) for unpublished literature. A total of 32 quantitative primary studies met all inclusion criteria and were included in the present review. Figure 1 details the results at each level and reasons for exclusion.

### Study Quality Assessment

**Fig. 2:**
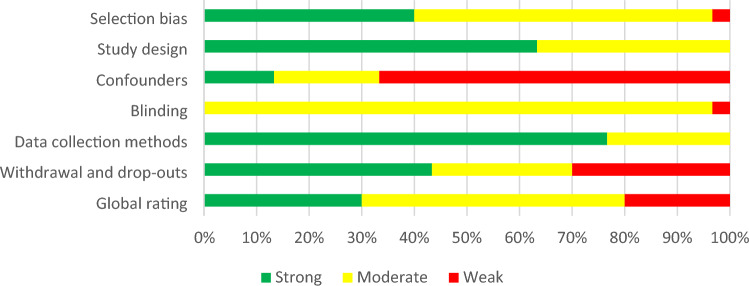
Visual representation of study quality

Overall, the quality of included published studies was moderate (*n* = 15, 50%); with some of high quality (*n* = 9, 30%) and the remaining of low quality (*n* = 6; 20%). The quality of included grey literature (*n* = 2; Koltz, 2022; Wahlund, 2022) was strong (low risk of bias). See Fig. 2 and Table 2 for a visual and tabular representation of study quality, respectively.

### Study Characteristics

Most studies were published studies (*n* = 30) and two were unpublished dissertations (Koltz, 2022; Wahlund, 2022). Table 3 provides a detailed description of included studies. All included studies reported on pre-post intervention outcomes, with 13 studies including additional follow-up assessments. Included studies predominantly followed a RCT study design (*n* = 18, 56%), with 11 single pre-post experimental studies (34%). 41% (*n* = 13) of studies included a single comparison group (active = 6; inactive = 7), while eight studies (25%) included two or more comparison groups which comprised of inactive controls and active controls.

Two studies reported on diverse populations. Schueller et al. (2019) included a sample of young people experiencing homelessness that were gender diverse or questioning. The intervention sample in Radovic et al. (2021) unintentionally included approximately one third (*n* = 6/20) of individuals who did not identify as male or female. Out of the 32 studies included, only 19% (*n* = 6) reported on gender diverse communities (e.g., non-binary) and/or sexual orientation. No study focused specifically on under-resourced communities or socioeconomics.

Studies were most commonly from the United States (*n* = 7, 22%) and Canada (*n* = 4, 13%). Three studies were from China, Finland, Germany, Netherlands (9%, respectively) and two from Australia, Italy, Sweden, United Kingdom (6%, respectively), while one study was from Indonesia (3%).

### Participant Characteristics

Included study sample size was highly variable, ranging from 4 to 5568 participants, with a mean sample size of 317. Excluding studies that did not report sample age range (*n* = 7), the mean participant age was 20.14 years (range 12–46). Eight studies included only participants aged ≥ 18 years. Study participants were predominantly female, with a mean of 72.28% female participants across studies (*n* = 29). All participants displayed emerging subclinical symptomatology.

### Intervention Characteristics

From the 32 included studies, we identified 29 unique brief digital mental health interventions that are guided (entirely or partially; i.e., ACT guide; BREATHE (6-module version); BREATHE (8-module version); BIP Worry; Entourage; ENJOY + Sense-It; ICare Prevent; Inroads; inSPIRE; I-BA; iSOMA; Tellmi; MIND; Moodpep; Pocket helper + Purple Chill + Slumber time; RESPOND; Rileks; SilverCloud;; Step-by-step; StudiCare-M; SOVA; UniWellbeing; WeChat mini; Youth COMPASS; Unnamed [*n* = 5]). Three interventions were reported on multiple times in separate studies: Youth COMPASS (Lappalainen et al., 2021, 2023), iSOMA-guided (Hennemann et al., 2022a, 2022b) and BREATHE (6-module version) (O'Connor et al., 2022; Radomski et al., 2020). Table 4 provides a detailed description of included interventions.

Intervention engagement period ranged from 20 days to 12 weeks (*M* = 7.34 weeks). Where reported, the average number of modules per intervention was 6.22 (range 3–12, *n* = 22 interventions), and the average number of modules intended to be completed per week of the intervention was 1.87 (range 1–6, *n* = 15 interventions). Mean number of sessions completed by study participants was 4.81 modules (*n* = 6 studies) and mean rate of completion (i.e., proportion of participants completing all modules) was 42.56% (*n* = 11 studies). Technology delivery mode was mixed: 14 interventions were web-based, four were mobile app-based (Ravaccia et al., 2022; Schueller et al., 2019; Sit et al., 2022; Sun et al., 2022), two via telehealth (i.e., Zoom/videoconferencing software; (Harra & Vargas, 2023; Novella et al., 2022)), and nine via a combination of delivery methods.

Of the 29 guided interventions, four (14%) offered solely human support, while 25 (86%) were partially guided and included a combination of human support and self-directed program elements. Twelve interventions offered human support via asynchronous methods, and four via synchronous contact. The remaining 13 interventions provided human support via a combination of asynchronous and synchronous methods. Of the interventions that were reported across multiple studies [*n* = 3 studies; Youth COMPASS (Lappalainen et al., 2021, 2023), iSOMA-guided (Hennemann et al., 2022a, 2022b) and BREATHE (O'Connor et al., 2022; Radomski et al., 2020)], in no cases were the human support methods compared. Mental health professionals were the primary providers of guided intervention content (*n* = 12 interventions, 43%), followed by interventions delivered by clinicians and psychology students together (*n* = 6 interventions, 21%), and peers (*n* = 3, Harra & Vargas, 2023; Klimczak et al., 2023; Rodriguez et al., 2021). Researchers were the sole human support for two interventions (Lappalainen et al., 2021; Sun et al., 2022). Together, peers and clinicians delivered guidance on two interventions (Rice et al., 2020; van Doorn et al., 2022) while clinical psychology students provided guidance in one intervention (Garnefski & Kraaij, 2023).

Regarding theoretical orientation, the most common intervention framework was cognitive behavioral therapy (CBT; *n* = 16 interventions), followed by acceptance and commitment therapy (ACT; *n* = 5 interventions), mindfulness (*n* = 3; Küchler et al., 2023; Rodriguez et al., 2021; Sun et al., 2022), and positive psychology models (*n* = 2; Schueller et al., 2019; van Doorn et al., 2022). Four interventions used multiple theoretical frameworks (Koltz, 2022; Küchler et al., 2023; Schueller et al., 2019; van Doorn et al., 2022). Two studies did not report on the therapeutic orientation of the intervention (Harra & Vargas, 2023; Ravaccia et al., 2022). A single intervention drew on the frameworks of ACT and mindfulness (Küchler, et al., 2023) while others drew on CBT and positive psychology (Schueller et al., 2019; van Doorn et al., 2022) or social cognitive models (Koltz, 2022).

### Socioemotional Outcome

Informed by observed outcome frequency, the primary mental health symptoms of examination included anxiety, depression, stress, wellbeing, mindfulness, and quality of life. Most studies (*n* = 29/31, 93.55%) examined several socioemotional outcomes. Only two studies examined one primary outcome (Koltz, 2022; Radomski et al., 2020). Anxiety and depression were the most common socioemotional outcome examined (*n* = 23; 72%, respectively), followed by stress (*n* = 10, 31%), well-being (*n* = 5, 16%), mindfulness (*n* = 3, 9%), and quality of life (*n* = 3, 9%).

#### Anxiety Symptoms

Twenty-three studies assessed anxiety symptoms, including three studies that assessed social anxiety (Novella et al., 2022; Rice et al., 2020; Stapinski et al., 2021) and one assessing academic anxiety (Radomski et al., 2020). Studies assessed anxiety symptoms via the GAD (*n* = 11), DASS-21 (*n* = 3; Juniar et al., 2020; Klimczak et al., 2023; Rodriguez et al., 2021), MASC (*n* = 3; O’Connor et al., 2020; O’Connor et al., 2022; Radomski et al., 2020), BAI (*n* = 2; Cerutti et al., 2022; Novella et al., 2022), STAI (*n* = 2; Lappalainen et al., 2023; Celia et al., 2022), SIAS (*n* = 2; Rice et al., 2020; Stapinski et al., 2021), CCAPS (*n* = 1; Novella et al., 2022), GRCS (*n* = 1; Radomski et al., 2020), SCID-I (*n* = 1; Cook et al., 2019), PSWQ (*n* = 1; Cook et al., 2019), MASQ (*n* = 1; Harra & Vargas, 2023), LSAS (*n* = 1; Rice et al., 2020), BFNE (*n* = 1; Rice et al., 2020), and ASI (*n* = 1; Rice et al., 2020). The number of intervention sessions ranged from 3 to 12 (*M* = 6.39). The efficacy of guided and partially guided digital delivery interventions in treating anxiety symptoms was compared to a control group(s) in 17 studies, 16 of which were RCTs and one of which was an experimental study (3-arm; Pescatello et al., 2021). Studies included either inactive controls (*n* = 7), active controls (*n* = 5), or a mix of both (*n* = 5).

A number of studies reported that intervention groups observed significantly greater short-to long-term anxiety symptom reductions when compared to either an inactive (Küchler et al., 2023; O'Connor et al., 2022; Radomski et al., 2020; Radovic et al., 2021) or active control group (Küchler et al., 2023; Stapinski et al., 2021; Sun et al., 2022, *p* values ≤ 0.001–0.43). In contrast, no significant differences between intervention and active/inactive control on anxiety outcomes were reported for many interventions (Cook et al., 2019; Harra & Vargas, 2023; Hennemann et al., 2022b; Karyotaki et al., 2022; Klimczak et al., 2023; Lappalainen et al., 2023; Novella et al., 2022; Pescatello et al., 2021; Rodriguez et al., 2021), demonstrating substantial heterogeneity across anxiety results.

Six studies conducted single-arm pre-post studies to examine intervention effects on anxiety symptoms. Five (83.33%) found a significant reduction in pre-post intervention anxiety symptoms (*p* < .001 to* p* = 0.024), while one study (Rice et al., 2020) found significant social anxiety reductions on various measures (LSAS; SIAS, BFNE; ASI; *p values* ≤ 0.001).

When contrasting studies that were entirely guided by a human support (*n* = 4; Celia et al., 2022; Cerutti et al., 2022; Harra & Vargas, 2023; Novella et al., 2022) to partially guided interventions (*n* = 19), it was found that half of the entirely guided interventions resulted in significant pre to post intervention anxiety declines (Celia et al., 2022; Cerutti et al., 2022), while the other half found non-significant group differences in pre to post intervention, or follow-up changes on various domains of anxiety, including social and generalized anxiety (Harra & Vargas, 2023; Novella et al., 2022). Partially guided interventions (*n* = 19) also found heterogenous results and only 53% (*n* = 10) of studies favored the intervention over control. Seven interventions were asynchronous, four were synchronous and eight were both. The use of synchronous or asynchronous guidance did not appear to influence anxiety outcomes.

For studies assessing anxiety symptoms, human support was provided in interventions by either mental health professionals (*n* = 10), mental health professionals and students together (*n* = 3; Pescatello et al., 2021; Radovic et al., 2021; Sit et al., 2022), researchers and students together (*n* = 2; Karyotaki et al., 2022; Lappalainen et al., 2023), peers (*n* = 3; Harra & Vargas, 2023; Klimczak et al., 2023; Rodriguez et al., 2021), researchers (*n* = 2; O'Connor et al., 2020; Sun et al., 2022), paraprofessionals or lay workers (*n* = 2; O'Connor et al., 2022; Radomski et al., 2020), or peers and mental health professionals together (*n* = 1; Rice et al., 2020). Support personnel did not appear to influence anxiety symptom outcomes.

#### Depression Symptoms

Of the 23 studies that assessed depressive symptoms, studies primarily assessed depressive symptoms via the PHQ-9 (*n* = 14), DASS-21 (*n* = 3; Klimczak et al., 2023; Rodriguez et al., 2021; Stapinski et al., 2021) and DEPS (*n* = 2; Lappalainen et al., 2023; Lappalainen et al., 2021). The average number of sessions was 6.22 (range 4–12). The efficacy of guided and partially guided digital delivery interventions in treating depression symptoms was compared to a control group(s) in 15 studies, 13 of which were RCTs. Control groups included inactive controls (*n* = 11) and active controls (*n* = 12).

While some interventions demonstrated significant reductions in depression due to intervention when compared to inactive controls, results are mixed. Three studies reported significantly greater depression symptom reduction due to intervention (Klimczak et al., 2023, *p* < 0.001; Harra & Vargas, 2023, *p* < 0.05; Küchler et al., 2023, *p* = 0.020–0.048). In contrast, five studies found non-significant differences between control and intervention group in symptom reduction immediately post intervention (Karyotaki et al., 2022; Lappalainen et al., 2023) or at follow-up periods (Cook et al., 2019; Karyotaki et al., 2022; Radovic et al., 2021). Peynenburg et al. (2022), identified significant pre-post intervention group differences (*p* = 0.06), yet these effects were not maintained at follow-up (1- and 3-month, *p* = 0.25–0.52).

Changes in depressive symptoms were inconsistent and depended markedly on how the study data were collected. For example, Grudin et al. (2022) observed significant pre-intervention to follow-up declines in clinician-rated depressive symptoms for both intervention and active control groups (*p’s* < 0.001), but not the inactive control (*p* = 0.077), whereas significant declines in self-rated or parent-rated depressive symptoms were observed for all groups (inactive control, intervention group, active controls; all *p’s* < 0.01) (Grudin et al., 2022).

If guided support was synchronous (*n* = 2; Cerutti et al., 2022; Harra & Vargas, 2023), we found reduced depression symptoms. If guided support was asynchronous (*n* = 12) or both synchronous and asynchronous (*n* = 9), results suggest mixed benefit in depressive outcomes. Human support was provided by either mental health professionals (*n* = 9); a combination of mental health professionals and students (*n* = 5); peers (*n* = 3; Harra & Vargas, 2023; Klimczak et al., 2023; Rodriguez et al., 2021); researchers and students (*n* = 2; Karyotaki et al., 2022; Lappalainen et al., 2023); researchers (*n* = 2; Lappalainen et al., 2021; Sun et al., 2022); psychology students (*n* = 1; Garnefski & Kraaij, 2023); or peers and mental health professionals (*n* = 1; Rice et al., 2020). Human support personnel did not appear to influence depression outcomes.

#### Stress Symptoms

Of the 10 studies that assessed stress symptoms, the DASS-21 was the most frequently used validated measure (27%, *n* = 2; Klimczak et al., 2023; Rodriguez et al., 2021). Remaining studies assessed stress via a heterogeneous array of measures (DT: Celia et al., 2022, ELEI: Cook et al., 2019, PASS: Koltz, 2022, PSS-4: Küchler et al., 2023, DASS-42: Juniar et al., 2022, PCL-5: Schueller et al., 2019, PSYCHOLOPS: Sit et al., 2022, and Dutch EMA: Van Doorn et al., 2022). When reported, the average number of sessions was 7 (range 5–12). All included studies that assessed stress were between-group designs and included a control. Of the 10 included studies, four were RCTs and included both inactive and active controls (Cook et al., 2019; Klimczak et al., 2023; Küchler et al., 2023) and active controls (*n* = 2; Rodriguez et al., 2021; van Doorn et al., 2022).

Five single-arm pre-post studies assessed the impact of an intervention on stress, again yielding inconsistent results. From pre- to post intervention, three studies (Celia et al., 2022; Juniar et al., 2022; Sit et al., 2022) reported a significant reduction in stress (*p* range < 0.001–0.005), whereas two studies (Koltz, 2022, *p* NR; Schueller et al., 2019, *p* > 0.50) reported non-significant changes in academic stress (Koltz, 2022) and general levels of stress (Schueller et al., 2019).

Partially guided interventions (*n* = 9) yielded mixed results in stress reduction, with three studies (Juniar et al., 2022; Klimczak et al., 2023; Sit et al., 2022) reporting a significant reduction in stress levels, especially among those with higher baseline stress (Cook et al., 2019), while five studies reported non-significant stress changes from pre to post intervention (Koltz, 2022; Rodriguez et al., 2021; Schueller et al., 2019; van Doorn et al., 2022) or to follow-up (Küchler et al., 2023).

Synchronous guided support (Celia et al., 2022) resulted in a significant reduction in perceived stress (*p* < 0.001); however, asynchronous guided support identified mixed results with some studies identifying a significant stress reduction (Cook et al., 2019; Juniar et al., 2022), while others observing no change (Koltz, 2022) or inconclusive results (Küchler et al., 2023). Providers of guided support varied, synchronous guidance or asynchronous guided support was generally delivered via mental health personnel alone (*n* = 4; Celia et al., 2022; Cook et al., 2019; Juniar et al., 2022; Küchler et al., 2023) while both asynchronous and synchronous guided support was delivered by a combination of mental health professionals and students (*n* = 2; Schueller et al., 2019; Sit et al., 2022), peers (*n* = 2; Klimczak et al., 2023; Rodriguez et al., 2021), mental health professionals alone (Koltz, 2022), and peer and mental health professionals combined (*n* = 1; Van Doorn et al., 2022). Support personnel did not appear to influence stress outcomes. There appeared to be no difference between synchronous and asynchronous intervention guidance and pre- and post intervention stress outcome change.

#### Wellbeing

Five studies assessed wellbeing as measured by the ORS (Ravaccia et al., 2022), WHO-5 (Küchler et al., 2023; Sit et al., 2022), SWLS (Celia et al., 2022), SWEMWBS (Rice et al., 2020), LSS (Rice et al., 2020), and ESS (Rice et al., 2020). The mean number of intervention sessions was 5.67 (range 5–7, *n* = 3) (Celia et al., 2022; Küchler et al., 2023; Sit et al., 2022). Mean intervention duration was 9.50 weeks (range 8–12, *n* = 5). Three studies provided asynchronous guided support (Küchler et al., 2023; Ravaccia et al., 2022; Rice et al., 2020), one study provided synchronous and asynchronous guided support (Sit et al. 2022) and one provided synchronous guided support (Celia et al., 2022). Most studies used a single-arm pre-post design to examine treatment effects on wellbeing (Celia et al., 2022; Ravaccia et al., 2022; Rice et al., 2020; Sit et al., 2022). One study was a RCT that included an inactive control and active control group (Küchler et al., 2023). Regarding theoretical framework that guided these study’s interventions, two studies used a CBT framework (Rice et al., 2020; Sit et al., 2022), Juniar et al. (2022) used transitional method, and Celia et al. (2022) used integrated mind–body approach.

Of the four single-arm pre-post studies, two studies found significant wellbeing improvements from pre- to post intervention on various wellbeing measures (*p* = 0.001, Celia et al., 2022; SWEMWBS: *p* < 0.001, WVS: *p* < 0.001, Rice et al., 2020), however no significant wellbeing change on the LSS measure was found (*p* = 0.580; Rice et al., 2020).

Regarding delivery method, results were inconsistent. There were significant differences between inactive controls and interventions that solely used asynchronous guided delivery after 4 weeks (*p* < 0.001), 8 weeks (*p* < 0.001) and 6-month (*p* = 0.016) follow-up (Küchler et al., 2023). When using asynchronous and synchronous intervention delivery, Sit et al. (2022) did not find a significant increase in subjective well-being (*p* = 0.208, *d* = 0.386). Ravaccia et al. (2022) used asynchronous delivery and found that improvements in wellbeing from pre to post for girls, was approaching significance (*p* = 0.05), but pre-post changes were non-significant for boys (*p* > 0.05). Overall effects were also non-significant (pre-intervention *M*(SD) = 5.07(2.58); post intervention *M*(SD) = 4.44(2.23),* p* NR; Ravaccia et al., 2022). Differences between synchronous and asynchronous guidance did not appear to influence wellbeing outcomes (Celia et al., 2022; Küchler et al., 2023; Ravaccia et al., 2022; Rice et al., 2020; Sit et al., 2022).

#### Mindfulness

Three studies assessed mindfulness as measured by the MAAS (Sun et al., 2022), FMI (Küchler et al., 2023), and the FFMQ (Rodriguez et al., 2021). No studies targeted mindfulness in isolation. The intervention duration ranged from 4–8 weeks (*M* = 5.3). Mindfulness sessions lasted 15–20 min (Rodriguez et al., 2021), 5–40 min (Sun et al., 2022), to 45–60 min (Küchler et al., 2023). Intervention elements included mindfulness-based exercises, awareness of the mind, and working with challenges or difficulties. All studies included homework tasks and audio recordings.

All studies were RCTs. Küchler et al. (2023) employed a RCT 3-arm method (with active and inactive controls) while Rodriguez et al. (2021) and Sun et al. (2022) employed a 2-arm RCT (with active control). Küchler et al. (2023) found mindfulness significantly improved after 4 weeks (*p* < 0.001), 8 weeks (*p* < 0.001) and 6-month follow-up (*p* < 0.001) in the intervention group (guided) compared to inactive control (waitlist). Similarly, significantly higher mindfulness was observed at 4 weeks (*p* < 0.001), 8 weeks (*p* < 0.001) and 6-month follow-up (*p* < 0.001) in the active control group (unguided) compared to inactive control (waitlist), suggesting that both mindfulness interventions (guided and unguided) were more efficacious compared to inactive control (waitlist). However, when comparing the intervention group (guided) to an active control (unguided), mindfulness did not significantly differ after 4 weeks (*p* = 0.56), 8 weeks (*p* = 0.90) and 6-month follow-up (*p* = 0. 08) (Küchler et al., 2023). Similarly, differences between intervention (mindfulness program with guidance) and active control groups (mindfulness program only with no guidance) on pre-post change in mindfulness was non-significant (Rodriquez et al., 2021; *p* = 0.53) suggesting that guidance did not significantly improve mental health outcomes. Both active control (social support-based intervention) and intervention (mindfulness-based intervention) improved on mindfulness from pre-post (Sun et al., 2022), with greater increases on mindfulness from pre to follow-up in the mindfulness-based intervention relative to social support intervention, however this was non-significant,* p* = 0.065 (Sun et al., 2022).

Küchler et al. (2023) and Sun et al. (2022) used asynchronous guided support and the other study (Rodriguez et al., 2021) was asynchronous and synchronous guided support. Both synchronous and asynchronous guidance did not appear to influence mindfulness outcomes. One study employed professional psychologist e-coaches to deliver the intervention (Küchler et al., 2023). Sun et al. (2022) used mindfulness trained research assistants and Rodriguez et al. (2021) used supervised and trained peer counselors. Support personnel did not appear to influence mindfulness outcomes.

#### Quality of Life

Three web-delivered studies assessed quality of life via the EQ-5D (Karyotaki et al., 2022), WHOQOL-BREF (Juniar et al., 2022), and YQOL-SF (O'Connor et al., 2022). Two of the included studies were two-arm RCTs (Karyotaki et al., 2022; O’Connor et al., 2022) and the remaining study (Juniar et al., 2022) was a single-arm feasibility pre-post design. Juniar et al. (2022) and Karyotaki et al. (2022) used psychologists to provide intervention guidance and O’Connor et al. (2022) employed research team members. Karyotaki et al. (2022) and Juniar et al. (2022) both provided asynchronous guided support, while O’Connor et al. (2022) used both asynchronous and synchronous guided support.

Both RCTs found no significant differences when comparing an intervention group to an inactive control. This was observed immediately post intervention (*p* > 0.05, Karyotaki et al., 2022) and at 3 to 12-month follow-up (Karyotaki et al., 2022,* p* > 0.05; O’Connor et al., 2022,* p* = 0.23). Juniar et al., (2022), via a single-arm design, identified significant pre- to post improvements in quality of life across various areas, including overall quality of life *(p* = 0.01), overall health *(p* = 0.03), physical health *(p* < 0.001), and psychological health (*p* = 0.003), with the exception of quality of life regarding social relationships (*p* = 0.45) and environmental health (*p* = 0.13).

### Reported Socioemotional Outcomes and Efficacy

Common DMHI elements associated with socioemotionally efficacious and non-efficacious interventions were explored. As above, results were separated by the following reported socioemotional outcomes: depression, anxiety, stress, wellbeing, mindfulness, and quality of life (see Table 5). A DMHI element was deemed efficacious if it reported a statistically significant effect on the socioemotional outcome under examination. Contrastingly, a DMHI element was deemed ineffective if no significant difference was found in that outcome, or if it was no different to a control condition. DMHI elements were reported when they were observed in two or more studies (*n* = 21). This was viewed as a preliminary exploration to examine potential associations and trends with intervention element and socioemotional outcome. Thus, findings do not imply the efficacy of a particular element and the identified elements may still be effective even if they are associated with treatment failure in this review.

**Table 5:** Key socioemotional outcomes of included studies

Study (year) Level of evidence	Outcome (measure)	Synchronous guided intervention delivery						
		Key findings						
		Depression	Anxiety	Stress	Well-being	Mindfulness	Quality of life	Other
Celia et al. (2022) **Level 5**	1. Anxiety (STAI-Y) 2. Stress (DT) 3. Global mental distress (YP-CORE) 4. Subjective well-being (SWLS) 5. Positive and Negative Affect Schedule (PANAS)		State (*p* = 0.002, *d* = 0.59) and trait anxiety (*p* = 0.003, *d* = .57) significantly ↓ post intervention	Perceived stress (*p* < 0.001, *d* = 0.66) significantly ↓ post intervention	Subjective wellbeing significantly ↑ pre to post (*d* = − 0.58,* p* = 0.001)			Global mental distress *p* < 0.001, *d* = 0.80 significantly ↓ post intervention Negative affect significantly ↓ post intervention* p* < 0.001, *d* = 0.66
Cerutti et al. (2022) **Level 5**	1. Depression (BDI-II) 2. Anxiety (BAI) 3. General functioning (OQ-45) 4. Hopelessness (BHS) 5. Personal burnout (CBI)	Significant pre-post ↓in depression. Mean change high (*d* = 1.11, *p* < 0.001)	Significant pre-post ↓in anxiety, with high mean change (*d* = 0.69, *p* < 0.001)					Significant pre-post improvement in general functioning, with high mean change (*d* = 0.70, *p* < 0.001). Significant pre-post ↓for hopelessness. Change low-moderate (*d* = 0.35, *p* < 0.01). Significant pre-post ↓for burnout. Change low-moderate (*d* = 0.46, *p* < 0.001)
Harra and Vargas (2023)* **Level 1**	1. Depression (PHQ-18) 2. Anxiety (MASQ)	Significant ↓ post intervention depression symptoms (*d* = 0.48, *p* < 0.05), with larger ↓ in treatment vs. control	NS group differences on anxiety symptoms across all domains (general distressed anxious symptoms: *d* = 0.04, general distress mixed symptoms: *d* = 0.53; anxious arousal: *d* = 0.13; general distress depressive symptoms: *d* = 0.08). All* p* values NR. Significant group differences on anhedonic depression subscale; *d* = 0.79, *p* < 0.05)					
Novella et al. (2022)* **Level 1**	1. Generalized anxiety (CCAPS) 2. Social anxiety (CCAPS) 3. Clinical anxiety (BAI)		NS difference in pre to f/u change in generalized anxiety (in-person *M* = − 7.29, SD = 6.71; online delivery *M* = − 6.38, SD = 4.79; *t*(39) = − 0.222, *p* = 0.640). NS difference in pre to f/u change on social anxiety (in-person *M* = − 4.312, SD = 4.14; online delivery *M* = *− *2.666, SD = 3.80; *t*(27) = − 1.161, *p* = 0.291). NS difference in clinical anxiety at pre, post and f/u between delivery systems (*F*(1, 33) = 0.313, *p* = .580, *n*_*p*_^1^ = 0.009). BAI post and f/u score significantly ↓than pre (*F*(1, 33) = 13.556, *p* = 0.001, *n*_*p*_^2^ = 0.001)					

Study	Outcome (measure)	Asynchronous guided intervention delivery						
		Key findings						
		Depression	Anxiety	Stress	Well-being	Mindfulness	Quality of life	Other
Cook et al. (2019)* **Level 1**	1. Depression (PSWQ; PHQ-9) 2. Anxiety (SCID-I, GAD, PSWQ) 3. Stressful events (ELEI) 3. Rumination (RRS) 5. Worry (PSWQ)	When controlling for past depression and baseline stress, 34% ↓ risk of depression in guided i-RFCBT vs. control, although NS (HR 0.66, *p* = 0.20). Guided i-RFCBT reduced depression risk by 34% vs. usual care (HR 0.66, *p* = 0.20). Significant improvements in depressive symptoms in short-to-medium term (statistics NR). Unguided i-RFCBT 36% ↓risk of future depression vs. control (HR 0.64, *p* NR). At 6-months, depression significantly ↓for guided i-RFCBT vs. control (*p* < .05). NS between-group differences at 3, 6, 15-month f/u (*ps* < 0.05)	NS between-group differences on anxiety symptoms at 3-, 6- and 15-month f/u (*p* > 0.05)	Participants with higher baseline stress benefited most from intervention (HR 0.43, *p* = 0.02) Unguided i-RFCBT larger effect for undergraduates with moderate-severe baseline stress (HR 0.48, *p* NR)				At 3-month f/u, rumination scores significantly lower for guided i-RFCBT vs. usual care (*p* < 0.05). NS group differences at 6 and 15-month f/u (*ps* > 0.05). At 6-month f/u, worry significantly lower for guided i-RFCBT vs. usual care (*p* < 0.05), and there was NS between-group differences at 3- and 15-month f/u (*p* < 0.05) for guided i-RFCBT and control
Hennemann et al. (2022a)* **Level 1**	1. Emotional reactivity (PERS) 2. Somatosensory amplification (SSAS) 3. Somatic symptom distress (PHQ-15)							Stronger pre-post improvements favoring iSOMA in somatic symptom distress, with medium effects (*d* = 0.70, *p* < 0 .001). NS moderating effect of Emotional Reactivity and treatment effects on somatic symptom distress (positive reactivity*: B* = − 0.08, *p* = 0.144; negative reactivity:* B* = − 0.02, *p* = 0.686). Somatosensory amplification moderated the outcome favoring iSOMA, i.e., higher pre-test somatosensory amplification associated with better outcome in active vs. control (*B* = − 0.17, *p* = 0.031). Main effect of treatment NS when SSAS as moderator and controlling for PHQ-15 pre-test scores (*B* = 2.43, *p* = 0.287). In WL group, ↑pre SSAS scores associated with ↑post PHQ-15 scores, as indicated by significant main effect of SSAS scores (*B* = 0.15, *p* = 0.009). Depression NS moderate effect of intervention vs. control on somatic symptom distress at post-assessment (*B* = − 0.54, SE = 0.12, *p* = 0.654). Anxiety NS moderate effect of intervention vs. control on somatic symptom distress post-assessment (*B* = − 0.02, SE = 0.11, *p* = 0.878)
Hennemann et al. (2022b)* **Level 1**	1. Depression (PHQ-9) 2. Anxiety (GAD-7) 3. Somatic symptom distress (SSD) 4. Therapeutic alliance (WAI-SR)	Significant pre-post ↓ in negative affect (iSOMA-guided: (*d* = 0.92, *p* = 0.370), iSOMA-GoD: *d* = 0.55) with NS group differences (*p* = 0.393) NS between-group difference in frequency of reliable change in PHQ-15 (*p* = 1.00)	NS (*p* = 0.335) effects of treatment conditions on anxiety iSOMA-guided: (*d* = 0.58), iSOMA-GoD: *d* = 0.48)					Significant pre-post ↓ in somatic symptom distress (iSOMA-guided: (*d* = 0.86), iSOMA-GoD: *d* = 0.63), with NS group differences (*p* = 0.467). iSOMA-guided group reached statistically significant change in somatic symptom distress compared to iSOMA-GoD (*p* = 0.011). Strong therapeutic alliance in both conditions, with NS difference between groups (iSOMA-guided: *M* = 3.38, SD = 0.83; iSOMA-GoD: *M* = 3.28, SD = 0.62, *p* > 0.05)
Juniar et al. (2022) **Level 5**	1. Depression (DASS-42) 2. Anxiety (DASS-42) 3. Stress (DASS-42) 4. Quality of life (WHOQOL-BREF)	Significantly ↓ depression pre-post (*M* = − 6.85, *d* = 0.58, *p* = 0.02)	Significantly ↓ anxiety pre-post (*M* = − 6.45, *d* = 0.62, *p* = 0.01)	Significantly ↓stress pre-post (*M* = − 10.04, *d* = 0.93, *p* < 0.001)			Significant ↑ quality of life for physical health (*d* = 1.25, *p* < 0.001), psychological health (*d* = 0.78, *p* = 0.003), overall quality of life (*d* = 0.62, *p* = 0.01), overall health (*d* = 0.54, *p* = 0.03) pre-post. NS differences in social relationship (*p* = 0.45) and environmental health domains of QoL (*p* = 0.13)	
Karyotaki et al. (2022)* **Level 1**	1. Depression (PHQ-9) 2. Anxiety (GAD-7) 3. Quality of life (EQ-5D) 4. Diagnoses of mental health disorders (MINI)	ITT analyses: NS differences between intervention and control depression symptoms post-test (*β* = − 0.50, *p* > 0.05), 6- (*β* = 0.30, *p* > 0.05) and 12-month f/u (*β* = − 0.19; *p* > 0.05) Complete-cases analyses: Similar results from ITT analyses were observed	ITT analyses: NS differences between intervention and control in anxiety symptoms at post-test (*β* = − 0.46, *p* > 0.05) and at 6- (*β* = 0.13; *p* > 0.05) and 12-month f/u (*β* = − 0.61; *p* > 0.05). Complete-cases analyses: Similar results from ITT analyses were observed				ITT analyses: NS differences between intervention and control QoL post-test (β = − 0.005, *p* > 0.05), 6-month (β = 0.01, *p* > 0.05) and 12-month f/u (β = 0.003; *p* > 0.05) Complete-cases analyses: Similar results from ITT analyses were observed. NS difference between guided iCBT and TAU in quality of life at post-treatment (β = − 0.005; *p* > 0.05) and f/u (6-months: β = 0.01; 12-months: β = 0.003; *p* > 0.05)	At 12-months, NS difference in depression and anxiety diagnoses between intervention and control (MDD: β = 0.12, GAD: β = − 0.62, SE = 1.0.; panic disorder: β = − 0.41, agoraphobia: β = − 0.022, *p* > 0.05)
Küchler et al. (2023)* **Level 1**	1. Depression (PHQ-9) 2. Anxiety (GAD-7) 3. Stress (PSS-4) 4. Well-being (WHO-5) 5. Mindfulness (FMI)	ITT analysis: Comparisons between UG vs. WL yielded mostly significant results after 4 wks (*B* = − 0.23, *p* = 0.048), 8 wks (*B* = − 0.33, *p* = 0.020) and 6- months (*B* = − 0.31 *p* = 0.045). Comparisons between GoD vs. WL yielded significant results after 4 wks (*B* = − 0.28, *p* = 0.025), 8 wks (*B* = − 0.44, *p* < 0.001) and 6- months (*B* = − 0.40, *p* = 0.007). Comparisons between GoD and UG yielded NS results after 4 wks (*B* = − 0.05, *p* = 0.706), 8 wks (*B* = − 0.11, *p* = 0.465) and 6-months (*B* = − 0.09, *p* = 0.62)	ITT analysis: Comparisons between UG vs. WL yielded mostly significant results after 8 wks (*B* = − 0.36, *p* = 0.014) and 6- months (*B* = 0.− 0.37, *p* = 0.012), with exception of 4 wks (*B* = − 0.22, *p* = 0.077). Comparisons between GoD vs. WL yielded significant results after 4 wks (*B* = − 0.46, *p* < 0.001), 8 wks (*B* = − 0.58, *p* < 0.001) and 6-months (*B* = − 0.66, *p* < 0.001). Comparisons between GoD and UG yielded mostly NS results after 4 wks (*B* = − 0.23, *p* = 0.082), and 8 wks (*B* = − 0.20, *p* = 0.166), however significant difference after 6-months (*B* = − 0.28, *p* = 0.026), where improvement significantly higher in GoD vs. UG	ITT analysis: Comparisons between UG vs. WL yielded mostly significant results after 4 wks (*B* = − 0.35, *p* = 0.008), 8 wks (*B* = − 0.31, *p* = 0.030) except at 6-months (*B* = − 0.23, *p* = 0.102). Comparisons between GoD vs. WL yielded significant results after 4 wks (*B* = − 0.47, *p* < 0.001), 8 wks (*B* = − 0.60, *p* < 0.001) and 6-months (*B* = − 0.47, *p* = 0.004). Comparisons between GoD and UG yielded mostly NS after 4 wks (*B* = − 0.12, *p* = 0.390), and 6-months (*B* = − 0.24, *p* = 0.155), with exceptions of stress at 8 wks (*B* = − 0.29, *p* = 0.031), where improvement significantly higher in GoD vs. UG	ITT analysis: Comparisons between UG vs. WL yielded mostly significant results after 8 wks (*B* = 0.40, *p* = 0.004) and 6-months (*B* = 0.42, *p* = 0.015), except for 4 wks (*B* = 0.20, *p* = 0.140). Comparisons between GoD vs. WL yielded significant results after 4 wks (*B* = 0.52, *p* < 0.001), 8 wks (*B* = 0.51, *p* < 0.001) and 6-months (*B* = 0.34, *p* = 0.016) Comparisons between GoD and UG yielded mostly NS results after 8 wks (*B* = 0.10, *p* = 0.508), and 6- months (*B* = − 0.08, *p* = 0.589), with exceptions of well-being at 4 wks (*B* = 0.32, *p* = 0.023), where improvement significantly higher in GoD vs. UG	Large effects (*d* = .94–1.07) post intervention when comparing both UG and GoD against WL Mindfulness significantly improved after 4 wks, 8 wks and 6-months in both intervention groups (IGs) compared with WL ITT analysis: Comparisons between UG vs. WL yielded significant results after 4 wks (*B* = 0.65, *p* < 0.001), 8 wks (*B* = 0.88., *p* < 0.001) and 6-months (*B* = 0.73, *p* < 0.001). Comparisons between GoD vs. WL yielded significant results after 4 wks (*B* = 0.76, *p* < 0.001), 8 wks (*B* = 0.88, *p* < *0.001*) and 6- months (*B* = 0.97, *p* < 0.001). Comparisons between GoD and UG yielded NS results after 4 wks (*B* = 0.07, *p* = 0.56), 8 wks (*B* = − 0.02, *p* = 0.90) and 6-months (*B* = 0.22, *p* = 0.80)		
Pescatello et al. (2021)* **Level 3**	1. Depression (PHQ-9) 2. Anxiety (GAD-7) 3. Treatment outcome (OQ-45)	SC-ONLY vs. SC + TX NS difference for depression (*b* = 0.20, *p* = 0.39). High severity participants using SC-ONLY vs. SC + TX NS depression difference (*b* = 0.20, *p* = 0 .77)., no group differences for participants who experienced greater symptom change (*b* = 0.17, *p* = 0.65. NS differences in usage for participants who experienced a large amount of change on PHQ-9 (*b* = 0.14, *p* = 0.74	SC-ONLY vs. SC + TX NS difference anxiety (*b* = 0.34, *p* = 0.15). High severity participants using SC-ONLY vs. SC + TX NS anxiety difference (*b* = 0.75, *p* = 0.11). no group differences for participants who experienced greater symptom change on anxiety (*b* = 0.46, *p* = 0 .42). NS differences in usage for participants who experienced a large amount of change on GAD-7 (*b* = 0.33, *p* = 0.52)					SC + TX better outcomes than TX-ONLY (*b* = 1.83, *p* = 0.04) when controlling for therapy type and presenting concern. Relationship held when controlling for race, gender, and treatment length. High severity participants in SC + TX NS different treatment outcomes than TX-ONLY (*b* = 3.87, *p* = 0.08)
Peynenburg et al. (2022)* **Level 1**	1. Depression (PHQ-9) 2. Anxiety (GAD-7) 3. Academic functioning (PAF) 4. Mental health disability (SDS) 5. Alcohol consumption (AUDIT) 6. Drug use (DUDIT)	Large pre-post ↓for depression (*d* = 1.28–1.48), with improvements maintained 1-month (*d* = 1.27–1.37) and 3-month f/u (*d* = 1.22–1.31). Main effect for MI intervention with ↓in depression symptoms (between-group *d* = 0.23, 95% CI − 0.01–0.47; *p* = 0.06) from pre-post treatment. Between-group differences were NS at the 1-month or 3-month f/u (*p* = 0.25, − 0.52). Main effects in favor of accessing the booster on depression (*p* = 0.09). Those who accessed booster had larger improvements in depression (between-group *d* = 0.31) at 3-month f/u	Large pre-post ↓for anxiety (*d* = 1.46–1.72), with improvements maintained at 1-month (*d* = 1.29–1.51) and 3-month f/u (*d* = 1.19–1.31). main effect for MI intervention with ↓in anxiety symptoms (between-group *d* = 0.25, 95% CI 0.02–0.49; *p* = 0.04) post. Between-group differences NS at 1-month or 3-month f/u (*p* = 0.57, − 0.60). Between-group effects (those assigned to the booster versus those who were not assigned to any) were NS for anxiety (*p* = 0.21) or SDS (*p* = 0.61) at 3-month f/u					For MI, small between-group effect post treatment, such that clients who received MI had larger improvements on mental health disability (SDS) than clients who did not receive MI (between-group *d* = 0.35). At 1-month (*d* = − 0.24 to 0.23) and 3-month f/u (*d* = − 0.20 to 0.27), differences no longer present, and there were large within-group effect sizes for improvements on SDS, regardless of factor (MI vs. booster) (*d* = 1.02–1.25) and 3-month f/u (*d* = 0.97–1.18). Between-group effects for MI and those who accessed MI with booster NS for mental health disability (*p* = .61) at 3-month f/u. NS between-group differences found for academic functioning at any of 3 time points (*p* = 0.48–0.75). Main effects in favor of accessing booster on academic functioning (PAF) (*p* = .02). Clients who accessed booster had larger ↑ perceived academic functioning (between-group *d* = 0.42) at 3-month f/u.No main effect found for MI for AUDIT (*p* = .35) or DUDIT (*p* = .49) post -measures not administered during f/u
Radovic et al. (2021)* **Level 1**	1. Depressive symptoms (PHQ-9) 2. Anxiety symptoms (GAD-7) 3. Emotional support (MOS-SS) 4. Parent adolescent communication (PACS) 5. General functioning (MAFS)	ITT analysis: From pre to 6 wks, ↓in depression in the EUC group compared with SOVA group (*p* = 0.09) Per-protocol analysis: NS differences between adolescents accessing the SOVA intervention and those who did not access it on depressive symptoms (*p* = 0.71)	ITT analysis: ↓in anxiety in EUC group compared with SOVA group (*p* = 0.04) Per-protocol analysis: NS differences between adolescents accessing the SOVA intervention and those who did not access it on anxiety symptoms (*p* = 0.42)					↑ social support in EUC group vs. SOVA from pre-6 wks post (*p* = .02). NS changes (pre-6 wks post) between SOVA and EUC for general functioning *p* = 0.95, family functioning *p* = 0.95, peer functioning *p* = 0.70, parent–child communication: (openness of communication *p* = .030; extent of communication *p* = 0.67). Per -protocol analysis comparing change scores between adolescents accessing SOVA vs. those who did not: NS differences on adolescent functioning subscales, except ↑in peer functioning in SOVA vs. EUC (*p* = 0.02). NS differences between the SOVA and those who did not access it on general functioning (*p* = 0.31), family functioning (*p* = 0.53), parent–child communication [openness of communication (*p* = 0.49), extent of communication (*p* = 40)], and social support (*p* = .99)
Ravaccia et al. (2022) **Level 5**	1. Welll-being (ORS) 2. Mental health empowerment (MHES)				NS differences in well-being in group and gender subgroup analyses pre-post (pre *M*(SD) = 5.07(2.58); post *M*(SD) = 4.44(2.23), *p* NR). In subgroup analysis with young females, overall well-being increased by 0.83 points, from 3.34/10 at pre to 4.17/10 at post, although the difference was NS: *t* = 1.97, *p* = 0.05. NS differences in group and subgroup analysis for young males (*effects* NR)			Subgroup analysis examining females showed ↑ patient activation levels pre-post (*t* = 2.15, *p* = 0.04), meaning participants knew how to look after their health more after the intervention
Rice et al. (2020) **Level 5**	1. Depression (PHQ-9, MDRS-22) 2. Wellbeing (WVS LSS, SWEMWBS, ESS) 3. Social connectedness (DSSI, UCLA, SCS, INQ) 4. Social anxiety (LSAS, BFNE, ASI, SIAS) 5. Self-compassion (SCS short) 6. Self-esteem (RSES) 7. Emotional regulation (ERQ) 8. Guilt and shame (PFQ2-B)	↓ depressive symptoms and suicidality pre-post (PHQ-9 full scale: *d* = 0.66, *p* < .001; suicidality item: *d* = 0.27, *p* = 0.026). NS pre-post change on the MDRS-22 (*d* = 0.30,* p* = 0.01)	Significant ↓ in social anxiety symptoms pre-post on the LSAS (*d* = 0.73, *p* < 0.001) and SIAS (*d* = 0.53; *p* < 0.001). 48.33% (*n* = 29) showing reliable improvement. NS improvements pre-post on social anxiety when measured with the BFNE (*d* = 0.37; *p* = 0.001) and ASI (*d* = 0.34; *p* = 001)		Significant ↑ wellbeing pre-post (SWEMWBS; *d *= 0.50, *p* < 0.001; WVS: d = 0.41, *p* < 0.001). NS change pre-post on the ESS (d = 0.07. *p* = 0.580)			Loneliness ↓ pre-post across all scales: (UCLA: *d* = 0.63, *p* < 0.001; DSSI: *d* = 0.50, *p* < 0.001; SCS: *d* = 0.63, *p* < 0.001; INQ—perceived burdensomeness: *d* = 0.48, *p* < 0.001; INQ – thwarted belongingness: *d* = 0.58, *p* < 0.001) NS self-compassion change pre-post (*p* = 0.003, *d* = 0.35) ↑ in self-esteem pre-post (*d* = 0.47, *p* < .001) NS change in emotion regulation subscales: reappraisal (*d* = 0.05, *p* = 0.691) and suppression (*d* = 0.08, *p* = .509) NS pre-post change in guilt and shame (*d* = 0.17, *p* = .145)
Sun et al. (2022)* **Level 1**	1. Depression (PHQ-9) 2. Anxiety (GAD-7) 3. Mindfulness (MAAS) 4. Emotional Suppression (Chinese ERQ ESS subscale)	Large depression ↓in both groups from baseline to f/u (*p* < 0.001*, d* = 1.46 and 1.10, for mindfulness and social support conditions, respectively). Size of depressive symptoms reduction over time NS different by condition (between group *d* = 0.36). Reductions in depressive symptoms in mindfulness mHealth group from baseline to f/u (73.7% to 17.3%) vs. social support mHealth group (71.9% to 34.0%) NS, *p* = 0.056. Condition × time effect NS for depression (*p* = 0.430)	Both groups ↓ anxiety symptoms from baseline to f/u (*p* < 0.001, ds = 1.40 and 0.68 for mindfulness and social support conditions, respectively). The mindfulness mHealth group experienced greater improvement [Condition × Time *p* = 0.024]. A stronger ↓ in mindfulness mHealth condition (↓ from 63.2% to 9.6%), compared to 57.9% to 27.7% for social support group (*p* = 0.020)			Both mindfulness-based and social support-based conditions improved in mindfulness and social support outcomes over time (time effect: *p* < 0.01). Mindfulness mHealth condition had large effect from baseline to f/u in improving mindfulness (*d* = 1.17) vs. control (*d* = 0.67). Condition × time effect NS, though there was a trend of improvement on mindfulness in the mindfulness mHealth condition (compared to social support condition), *B* = 1.97, *p* = 0.065		Both mindfulness-based and social support-based conditions improved in social support outcomes over time (time effect: *p* < 0.01). Small effects for improvements in social support for control (*d* = 0.33) and mindfulness conditions (*d* = 0.10). Condition × time effect was NS for social support (*p* = 0.084) NS between-group difference emotional suppression change during intervention, *p* = 0.091. Emotional suppression ↓ from baseline to post linked to ↓ of depression and anxiety symptoms from baseline to f/u in mindfulness condition, opposite direction found in control
Wahlund (2022) **Level 5**	1. Depression (NR) 2. Anxiety (NR) 3. Worry (PSWQ-C) 4. Impaired functioning (NR)	Post-treatment significant medium to large ↓ in depressive symptoms (*d* = 0.69–1.38, *p* = 0.001). Post parent-reported depression significantly ↓ (*d* = 0.49–1.76*; p* = .001) with changes maintained at 1 and 3-month f/u	Post-treatment significant medium to large ↓ in anxiety symptoms (*d* = 0.69–1.38, *p* = 0.001). Post parent-reported significantly ↓anxiety (*d* = 0.49–1.76*; p* = 0.001) with changes maintained at 1 and 3-month f/u					Post-treatment medium to large significant↓ self-rated worry (*d* = 0.69–1.38, *p* = 0.001). Similar changes for depression reported by parents (*d* = 0.49–1.76*; p* = 0.001) and changes were maintained at 1 and 3-month f/u. Post-treatment results showed medium to large ↓ in impaired functioning (*d* = 0.69–1.38, *p* = 0.001)

Study	Outcome (measure)	Mixed synchronous *and* asynchronous guided intervention delivery						
		Key findings						
		Depression	Anxiety	Stress	Well-being	Mindfulness	Quality of life	Other
Garnefski and Kraaij (2023) Level 5	1. Depression (PHQ-9)	Post-test, 17/23 (73.91%) showed categorical improvements, 6/23 (26.09%) remained in same ‘cut-off’ category & 0 deteriorated. Post-test, 19/23 (82.61%) scored in one of the categories of minimal or mild depression, vs. 5/23 (21.74%) at pre-test. Significant prepost improvements on depression for Completers = 5.52 (*d* = 1.31) and ITT (started but did not complete) = 4.10 (*d* = 0.94)						
Grudin et al. (2022)* **Level 1**	1. Assessor-rated child depressive symptoms (CDRS-R) 2. Self-rated depressive symptoms (SMFQ-A) 3. Parent-rated depressive symptoms (SMFQ-P) 4. Impaired functioning (WSAS-A)	Significant ↓ in assessor-rated child depressive symptoms from pre to 3-month f/u for therapist-guided I-BA (*B* = − 11.3, *p* < 0.001) & self-guided I-BA (*B* = − 10.38, *p* < 0.001), but not TAU (*B* = − 4.40, *p* = 0.077*, p* > 0.05). Assessor-rated child depressive symptoms within-group *d* = 2.43 for therapist-guided I-BA, 2.23 for self-guided I-BA and 0.95 for TAU. Significant ↓ in self-rated depressive symptoms for all groups: therapist-guided I-BA (*B* = − 4.4, *p* < 0.001,), self-guided I-BA (*B* = − 3.39, *p* < 0.05) and TAU (*B* = − 4.04, *p* = 0.001,). Self-rated depression within-group effect *d* = 1.45 for therapist-guided I-BA, *d* = 1.12 for self-guided I-BA and *d* = 1.34 for TAU. Significant ↓ for parent-rated depressive symptoms for therapist-guided I-BA (*B* = − 2.83, *p* < 0.01), self-guided I-BA (*B* = − 3.75, *p* < 0.01), and TAU (*B* = − 3.29*, p* < 0.01). Parent-rated depression within-group *d* = 1.05 for therapist-guided I-BA, *d* = 1.40 for self-guided I-BA, *d* = 1.22 for TAU						Significant ↓for self-rated impaired functioning (WSAS-A) for therapist-guided I-BA (*B* = − 5.24, *p* < 0.001) and self-guided I-BA (*B* = − 3.58, *p* < 0.01), but not TAU (*B* = − 1.81, *p* = 0.163). For impaired functioning, within-group *d* = 1.47 for therapist-guided I-BA, 1.00 for self-guided I-BA and 0.51 for TAU
Keinonen et al. (2021) **Level 5**	1. Depression (DEPS) 2. Avoidance and cognitive fusion (AFQ-Y) 3. Perceived health (HBSC)	Depression ↓during 5-wk intervention for high symptoms youth (*B* = 1.76, *p* < 0.001). NS effects on depression for youth with average or stable symptoms (*p* > 0.05). Heightened depression for those with low experiential avoidance and decreasing depressive symptoms post intervention (*B* = − 0.64, *p* < 0.001)						Experiential avoidance ↓ during 5-wk intervention among those with high symptomatology (*B* = 1.73, *p* < .001). NS effects on experiential avoidance for those with average and stable symptoms (*p* > .05). Heightened experiential avoidance for those with low experiential avoidance and decreasing depressive symptoms (*B* = − 0.63, *p* < 0.001). Those in high and decreasing experiential avoidance and depressive symptoms trajectory perceived their health significantly ↓ (*p* < 0.001) and sleep (*p* < 0.001), ↑substance abuse (*p* = 0.001) and ↓physical activity (*p* = 0.035) pre-intervention than those from other two trajectories (1: Average and stable experiential avoidance and depressive symptoms; 2: Low experiential avoidance and decreasing depressive symptoms)
Klimczak et al. (2023)* **Level 1**	1. Depression (DASS-21) 2. Anxiety (DASS-21) 3. Stress (DASS-21) 4. Total psychological distress (MHC-SF) 5. Positive mental health (DASS-21) 6. Psychological inflexibility (AAQ-II) and flexibility (CompACT) 8. Openness to experience (CompACT) 9. Behavioral awareness (CompACT) 10. Valued action (CompACT)	Phone coaching experienced ↑ gains than control on depression (*p* = 0.035) pre-post. Text coaching NS effect on depression (*p* > 0.05). NS differences between phone and text coaching groups (*p* > 0.05). Assigned condition had significant effect on reliable improvement in depression (*X*^2^(2) = 15.6, *p* < 0.001). Phone condition significantly more likely to experience reliable improvement in depression (33%; *p* < 0.001) than control. NS differences between phone and text conditions, or text and control conditions (all *p* > 0.05)	Phone coaching ↑ gains vs. control for anxiety (*p* = 0.025) pre-post. Text coaching NS effect on anxiety (*p* > 0.05). NS differences were found between phone and text coaching groups (*p* > 0.05) Assigned condition had NS effect on reliable improvement in anxiety (*p* NR; % Reliable improvement for phone = 17%, text = 10%)	Phone coaching ↑ gains than control for stress (*p* = 0.045) pre-post. Text coaching NS effect on stress (*p* > 0.05). NS differences between phone and text coaching groups (*p* > 0.05). Assigned condition significant effect on reliable improvement in stress (*X*^2^(2) = 7.8, *p* = 0.021). Phone condition significantly more likely to experience reliable stress improvement (28%; *p* = 0.023) than control. NS differences between phone and text, or text and control groups (*ps* > 0.05)				Phone coaching ↑ gains vs. control pre-post for psychological distress (*p* = 0.007), positive mental health (*p* = .006), psychological inflexibility (*p* = 0.032), openness to experience (*p* < 0.001), behavioral awareness (*p* < 0.001), psychological flexibility (*p* < 0.001). NS difference between phone coaching and control for valued action (*p* = 0.134). Those receiving text message coaching experienced improved openness to experience (*p* = 0.025), behavioral awareness (*p* = 0.035), and psychological flexibility (*p* = 0.035) compared to control. Text coaching had no significant effect on psychological distress, positive mental health, psychological inflexibility, or valued action (all *p* > 0.05). NS differences between phone and text coaching groups (all *p* > 0.05). Assigned condition had significant effect on reliable improvement in psychological distress (*X*^2^(2) = 9.3, *p* = .009), positive mental health (*X*^2^(2) = 8, *p* = .018), psychological inflexibility (X^2^(2) = 7.6, *p* = .023). phone condition significantly more likely to experience reliable improvement in psychological distress (57%; *p* = .007), psychological inflexibility (20%; *p* = .018) compared to control. NS differences between phone and text conditions, or the text and control conditions (all *p* > 0.05)
Koltz (2022) **Level 5**	1. Perceived academic stress (PASS)			NS effects of online counseling on academic stress (P1: *d* = − 1.78*;* P2: *d* = − 0.44; P3: *d* = 0.12; P4: *d* = 0.66; (*ps* NR)				
Lappalainen et al. (2021)* **Level 1**	1. Depressive symptoms (DEPS) 2. Life satisfaction (SWLS) 3. Avoidance & cognitive fusion (ATQ-Y)	ITT analyses: change in interventions groups NS vs. control (*p* = 0.153). Changes for two iACT intervention groups significantly different vs. control (*d* = 0.16, *p* = 0.024). Depression symptoms ↓significantly more in both iACTface group (*d* = 0.15, *p* = 0.021) and iACT group (*d* = 0.16, *p* = 0.017) vs. control. Between-group effects small (*d* = 0.20, *p* < 0.05). Within-group pre-post change significant for both iACT groups, but not control (*d* = .05, *p* NR). Change in depressive symptoms equal in both intervention groups (*p* = 0.935). iACTface intervention, including two f2f meetings, ↓depression among girls but not boys (*p* = 0.006). NS gender differences for intervention including only support via WhatsApp (iACT) *p* > 0.05						ITT analysis: NS differences in changes in two iACT interventions compared to control for life satisfaction (*p* = 0.195). Intervention effect significant for life satisfaction (*d* = 0.30, *p* = 0.030). Life satisfaction ↑significantly more in iACT without f2f meetings vs. control (*d* = 0.04, *p* = 0.013). iACTface positive impact on life satisfaction, but difference vs. control NS (*p* = 0.065). For life satisfaction, within-group effect pre-post significant for both intervention groups (*p* < 0.05), but not control (*p* > 0.05). For life satisfaction, vs. control, between-group effect small. NS difference in changes in life satisfaction between two iACT interventions (*p* = 0.456). NS gender differences on life satisfaction in either intervention groups (*p* = 0.397). NS effect for avoidance (psychological flexibility), but small ↓in avoidance in intervention groups (iACTface: *d* = 0.03, iACT: *d* = 0.15) vs. small ↑in control (*d* = 0.06). NS differences in changes for avoidance between groups (*p* > 0.05). iACTface intervention, including two f2f meetings, ↓ avoidance of unpleasant feelings in girls not boys (*p* = 0.033). NS gender differences for intervention incl. only support via WhatsApp (iACT) *p* = 0.555)
Lappalainen et al. (2023)* **Level 1**	1. Depression (DEPS) 2. Anxiety (STAI) 3. Psychological flexibility (CompACT) 4. Self-compassion (SCS-SF)	ITT analysis: Changes in both intervention groups (iACT student coach + virtual coach; iACT virtual coach) NS difference to control on depressive symptoms (*p* = 0.179). Per-protocol analysis: Depression showed ↑in iACT group, but NS (*d* = − 0.01, *p* = 0.224)	ITT analysis: Changes in both intervention groups (iACT student coach + virtual coach; iACT virtual coach) NS different to control on anxiety (*p* = 0.073). Anxiety slight ↑in iACT group (within ES, *d* = 0.05, *p* = 0.042), anxiety symptoms in control significantly greater ↑ (within ES, *d* = 0.34, *p* NR)					ITT analysis: Changes in both intervention groups (iACT student coach + virtual coach; iACT virtual coach) did not NS differ to control for psychological flexibility (*p* = 0.421) and self-compassion (*p* = 0.112). iACT group showed different change (slight ↑) compared to control group for self-compassion (*d* = 0.12, *p* = 0.030). Psychological flexibility, but not self-compassion, predicted depression symptom changes (*F*(1,69) = 5.911, *p* = 0.18)
O'Connor et al. (2020)* **Level 1**	1. Anxiety (MASC-2) 2. Healthcare use (NR)		Experimental group: *M* change in anxiety baseline to 8-wks − 7.9 (SD = 15.7; *p* value NR). 80% CI for SD generated for 8-wks post to baseline change score 12.6 to 21.7. For control, *M* change in anxiety scores from 8-wks post to baseline − 9.0 (SD = 15.4; *p* NR). Difference of 4.7 in change scores between control and experimental group (*p* NR)					39% (14/36) reported using healthcare resources during BREATHE
^O'Connor et al. (2022)* **Level 1**	1. Anxiety (MASC-2) 2. Quality of life (YQOL-SF) 3. Healthcare use (trial-specific measure)		Post intervention, Δ = 5.5 difference between intervention groups (favoring online CBT; *p* = 0.019) estimated linear regression effect size of *B* = 0.32 (effect measurement NR). 3-month f/u, significant anxiety difference between-group mean difference of *M* = − 4.39 (favoring online CBT; *p* = 0.04)				At 3-month f/u, NS between-group differences in quality of life (*p* = 0.23)	Post, intervention group had fewer visits to psychiatrist *(%*Δ = –41%), social worker *(%*Δ = –42.5%), hospital-based healthcare *(ED visits: %*Δ –80%; hospital admission: %Δ –76.1%). Intervention group fewer self-help and alternative treatments (%Δ = –60%). Greatest change for static website group was fewer social worker visits (%Δ = –22.1%) and hospital-based healthcare visits *(ED visits: %*Δ = –79.4%; hospital admission: %Δ = –42.9%; *p* NR)
Radomski et al. (2020)* **Level 1**	1. Anxiety (MASC-2, GRCS)		NS relation between number of completed sessions and anxiety change on GRCS (rho = 0.02; *p* = 0.83). With the GRCS, 75% (60/80) improved anxiety post program (M improvement = 2.3 (‘somewhat better’). On MASC-2, MΔ = 13.8 (SD = 18.1). 43% (35/81) of intervention participants were positive treatment responders based on minimal clinically important difference (MCID) threshold. Significant differences between BREATHE and control on all anxiety items (*ps* < 0.001) with greater improvements in BREATHE intervention					
Rodriguez et al. (2021)* **Level 1**	1. Depression (PHQ-9, DASS-21) 2. Anxiety (GAD-7, DASS-21) 3. Stress (DASS-21) 4. Mindfulness (FFMQ)	NS pre-post depression change as measured on the DASS* p* = 0.41; *d* = 0.24. NS pre-post-depression change as measured on the PHQ (*p* = 0.26; *d* = 0.33) MIND + significantly greater pre-post depression improvements (interaction estimate = 0.38, SE = 0.16; *t*_330_ = 2.37; *p* = 0.02) than MIND group	Pre-post change on anxiety for both groups was NS on GAD and DASS: *p* = 0.80; *d* = − 0.07; *p* = 0.72; *d* = 0.10, respectively. Pre-post effect size for anxiety was large (*d* = 0.89)	Pre-post change for both groups NS on DASS: *p* = 0.76; *d* = 0.09. MIND + significantly greater pre-post improvements in daily stress ratings than MIND (interaction estimate = 0.39, SE = 0.18; *t* = 2.29; *p* = 0.02)		Pre-post mindfulness change for both MIND and MIND + groups NS (*p* = 0.53; *d* = 0.18)		
Schueller et al. (2019) **Level 5**	1. Depression (PHQ-9) 2. PTSD symptoms (PCL-5) 3. Emotional regulation (DERS)	NS pre-post-depression (*d* = 0.27, *p* > .50). Having no traumatic experience during intervention NS change on depressive symptoms (Δ = 0.33, *p* NR). Those who experienced a traumatic event throughout intervention period had small ↓in depressive symptoms (*M* Δ = 2.25), *p* = 0.30, *d* = − 0.49,		NS pre-post PTSD change (*d* = 0.17, *p* > 0.50). Having no traumatic experience during intervention ↓ PTSD symptoms (MΔ = 6.42, *p* NR), and poorer emotion regulation from pre to post. Those who experienced a traumatic event ↑ PTSD symptoms (MΔ = 3.78, *p* = 0.35, *d* = 0.42				NS pre-post emotion regulation change (*d* = 0.10, *p* > .50). Those who experienced a traumatic event had poorer pre-post emotion regulation (Δ = 1.00, *p* NR). Those who experienced a traumatic event throughout course of intervention had small ↑ in emotion regulation (Δ = 3.89), *p* = .63, *d* = − 0.22
Sit et al. (2022) **Level 5**	1. Depressive symptoms (PHQ-9) 2. Anxiety symptoms (GAD-7) 3. Self-defined stress (PSYCHOLOPS) 4. Wellbeing (WHO-5)	Significant ↓ depression scores post intervention (*t*(11) = 4.29, *p* = 0.001, *d* = 1.24)	Significant pre-post ↓ anxiety symptoms (*p* = 0.024, *d* = 0.754)	Significant pre-post ↓ self-defined stress (*p* = 0.005, *d* = 0.99)	NS pre-post ↑ subjective well-being (*p* = 0.208, *d* = 0.386)			
Stapinski et al. (2021)* **Level 1**	1. Anxiety (GAD-7) 2. Social anxiety (SIAS + SPS) 3. Depression (DASS-21) 4. Functional impairment (SDS)	Depression symptoms ↓ 2-month f/u for both groups (Inroads: *d* = 0.91, *p* < 0.001; control: *d* = 0.50, *p* < 0.001), weak evidence for greater ↓ for Inroads (*d* = 0.39, *p* = 0 .049). Ongoing ↓ in depression, with control (*d* = 0.71, *p* < 0.001) achieving comparable gains as Inroads by 6-month f/u (*d* = 0.96; *p* < 0.001)	Inroads & control significant ↓ general anxiety. Group × time interaction significantly greater ↓ 2-month f/u for Inroads (*d* = 0.88, *p* = 0.002). By 6-month f/u, control comparable ↓to Inroads, with no group differences (*d* = 0.38, *p* = .238). Social anxiety symptoms ↓ at 2-month f/u for Inroads (*d* = 0.48, *p* < 0.001) but not control (*d* = 0.14, *p* = .196). Inroads, but not control (*d* = 0.22; *p* = .080), significant ↓ social anxiety symptoms at 6-month f/u (*d* = 0.59; *p* < 0.001). Significant group x time interaction with Inroads vs. control at 2-month (*d* = 0.32, *p* = .045) and 6-month f/u (*d* = 0.37, *p* = 0 .043)					Functional impairment ↓for both groups at 2-month (Inroads: *d* = 0.52, *p* = .002; control: *d* = 0.79,* p* < 0.001) and 6-month f/u (control: *d* = 0.75, *p* < 0.001; Inroads: *d* = 1.01, *p* < 0.001). NS group x time interaction at 2-months (*d* = 0.28, *p* = .202) or 6-months (*d* = 0.22, *p* = 0.348). At 6-month f/u Inroads and control reported greater ↓in number of days lost (*b* = 0.58, *p* = .038; *d* = 0.31) and number of unproductive days due to symptoms (*p* = 0.022; *d* = 0.46)
van Doorn et al. (2022)* **Level 3**	1. Stress (Dutch EMA) 2. Emotional awareness (Dutch S-DERS)			NS effects on perceived stress post intervention (*B* = − 0.020, *p* = 0.562)				Significant ↑ in emotional awareness pre to post intervention (*B* = 0.030, *p* = 0.048). Significant time x condition interaction (*B* = 0.030, *p* = 0.048), indicating in experimental condition emotional awareness significant ↑ over time

^Async or sync depending on participant’s preference; ^ Comparative study; * RCT; ≠ No association; ↑ Increase; ↓ Decrease; Δ Average rate of change (delta), *ɳ*^*2*^ Eta squared, *ɳp*^*2*^ Partial eta squared, *AD* Anderson-Darling goodness of fit, *AA* anxious arousal, *b/B/*β beta, *CC* concentration capacities, *CI* confidence interval, *d* Cohen’s d effect size, *ES* effect size,* F* F ratio, *f/u* follow-up, *HR* hazard ratio, *ITT* intention-to-treat, *M* mean, *MM* mindfulness meditation, *NR* not reported, *NS* not significant, *P* participant, *p* probability value, *ps* probability values, rho Spearman’s correlation coefficient, *SD* standard deviation, *SE* standard error, *SC* SilverCloud, *t* t value, *TX* treatment only, *PTSD* post traumatic stress disorder, *vs.* versus, *WL* wait list, *X*^2^ chi square. Level of evidence: Level 1 = Studies described as randomised controlled trials; Level 2 = described as controlled study; Level 3 = Cohort analytic (two group pre + post); Level 4 = Case control; Level 5 = Cohort (one group pre + post (before and after). Measures: *AAQ-II* acceptance and action questionnaire-2nd version, *AFQ-Y* avoidance and fusion questionnaire for youth, *ASI* anxiety sensitivity index, *ATQ-Y* avoidance and fusion questionnaire for youth, *AUDIT* alcohol use disorders identification test, *BAI* beck anxiety inventory, *BDI-II* beck depression inventory-2nd version, *BFNE* brief fear of negative evaluation from others scale, *BHS* beck hopelessness scale, *CBI* Copenhagen burnout inventory, *CCAPS* counseling center assessment of psychological symptoms, *CompACT* comprehensive assessment of acceptance and commitment therapy, *DASS-21/42* depression anxiety and stress scale-21/42 item, *DEPS* depression scale, *DSSI* Duke social support index, *CDRS-R* children’s depression rating scale-revised, *DT* distress thermometer, *DUDIT* drug use disorders identification test, *ELEI* episodic life event interview, *EMA* ecological momentary assessment, *ERQ* emotional regulation questionnaire, *ESS* European Social Survey, *EQ-5D* EuroQol-5 digit health status, *FFMQ* five facet mindfulness questionnaire, *FMI* Freiburg mindfulness inventory, *GAD* generalized anxiety disorder, *GAD-7* generalised anxiety disorder-7 item, *GRCS* global rating of change scale, *HBSC* health behavior in school-aged children, *INQ* interpersonal needs questionnaire, *LSAS* life skills assessment scale, *LSS* life satisfaction scale, *MAAS *mindful attention awareness scale, *MAFS* multidimensional adolescent functioning scale, *MASC-2* multidimensional anxiety scale for children-2nd version, *MASQ* mood and anxiety questionnaire, *MDRS-22* male depression risk scale-22 item, *MINI* mini international neuropsychiatric interview, *MHC-SF* mental health continuum short form, *MHES* multidimensional home environment scale, *MOS-SS* medical outcomes social support survey, *ORS* outcome rating scale, *OQ-45* outcome questionnaire-45 item, *PACS* parent-adolescent communication scale, *PAF* perceptions of academic functioning, *PANAS* positive and negative affect schedule, *PASS* perceived academic stress scale, *PERS* Perth emotional reactivity scale, *PCL-5* posttraumatic stress disorder checklist for DSM-5, *PFQ2-B* personal feelings questionnaire 2-brief, *PHQ-9* patient health questionnaire-9 item, *PHQ-15* patient health questionnaire-15 item, *PSYCHOLOPS* psychological outcome profiles, *PSS-4* perceived stress scale-4 item, *PSWQ* Penn State Worry Questionnaire, *RRS* Rumination Response Scale, *RSES* Rosenberg self-esteem scale, *SCID-I* structured clinical interview for DSM disorders-Axis I disorders, *SCS* self-compassion scale, *SCS-SF* self-compassion scale short form, *S-DERS* state difficulties in emotion regulation scale, *SDS* sheehan disability scale, *SIAS* social interaction anxiety scale, *SMFQ-A* short mood and feelings questionnaire-adolescent version, *SMFQ-P* short mood and feelings questionnaire-parent report version, *SPS* suicide probability scale, *SSAS* somatosensory amplification scale, *SSD* somatic symptom distress, *STAI* state trait anxiety inventory, *STAI-Y* state trait anxiety inventory-youth version, *SWEMWBS* short Warwick Edinburgh Mental Well-Being Scale, *SWLS* satisfaction with life scale, *UCLA* University of California Los Angeles Loneliness Scale, *WAI-SR* work alliance inventory-short revised, *WEMWBS* Warwick-Edinburgh Mental Well-being Scale, *WHO-5* World Health Organisation-5 Well-being Index, *WSAS-A* work and social adjustment scale-adolescent version, *WVS* World values survey, *WHOQol-BREF* World Health Organization Quality of Life-Brief version, *YP-CORE* young person’s clinical outcomes in routine evaluation, *YQoL-SF* youth quality of life instrument-short form

Preventative interventions primarily focus solely on the immediate program period and do not provide ongoing support post intervention (*n* studies = 27). This has resulted in intervention effects that are not enduring long-term (Cook et al., 2019; Stapinski et al., 2021).

### Overall Efficacy of Socioemotional Outcomes Examined

The efficacy of DMHIs on youth socioemotional outcomes shows notable inconsistencies across various study designs. Positive impacts on depression, anxiety, and stress were observed in single-arm pre-post study designs. However, when compared to control groups in multi-arm studies, such as randomized controlled trials, the results were mixed for these same outcomes Additionally, there was evidence of poor or inconclusive improvements in certain areas: mindfulness outcomes in multi-arm studies and quality of life in both single-arm and multi-arm studies. Similarly, limited effectiveness was noted for wellbeing in single-arm studies.

#### Elements Common to DMHI with Established Efficacy

For DMHIs that were effective at enhancing socioemotional outcomes, common interventions elements include a combination of content delivery and activities (such as goal setting or emotion regulation) and program structure, such as follow-up support and participant feedback (See Table 6). Elements such as refresher/follow-up content, goal setting, and relapse prevention were common features of DMHIs that were efficacious for depression and anxiety, while content personalization and personalized recommendations were features of DMHIs that were efficacious for depression and stress. For the socioemotional outcomes of mindfulness and quality of life, no associated common DMHI elements were identified.

**Table 6:**
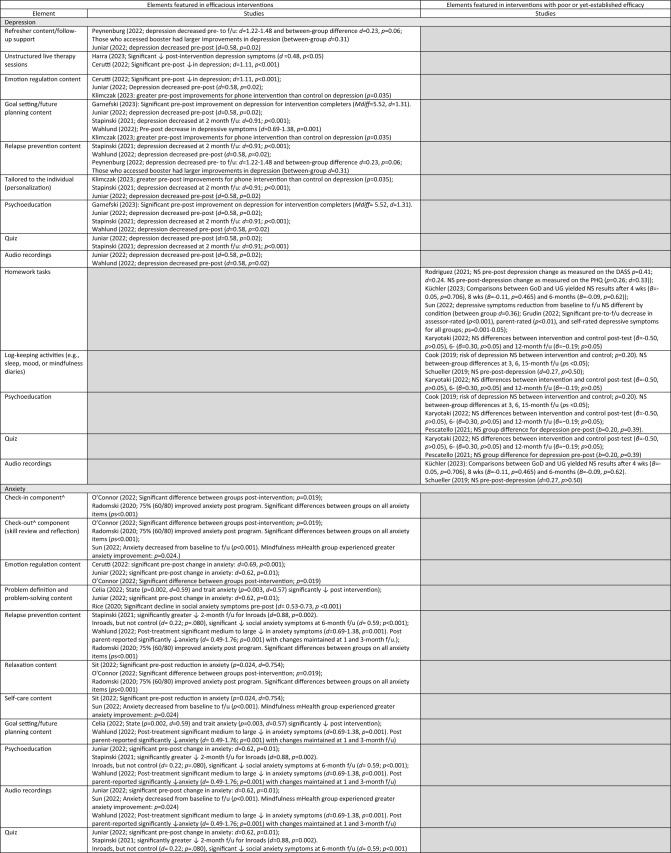

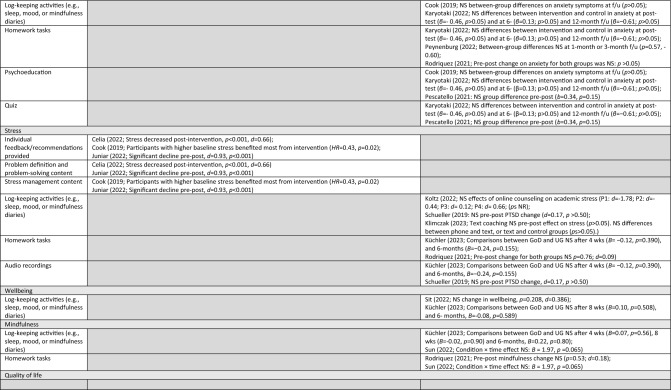
Common intervention elements and associated socioemotional outcomes

^**Check-in** served as a risk management strategy and involved adolescents rating their mental health over past week and whether they thought about harming themselves or others. In the event that a safety concern was flagged, research member contacted the adolescent (and potentially parent(s)) by phone within 36 h to assess whether more immediate care and resources required. **Check-out** involved adolescents engaging in self-reflection to session content

Dark grey shading = no data identified

*DASS* depression, anxiety and stress scale, *f/u* follow-up, *GoD* guidance on demand, *NS* non-significant, *PHQ* patient health questionnaire, *UG* unguided, *wks* weeks

#### Elements Common to DMHI with Poor or Yet Established Efficacy

For DMHIs that did not report significant findings in various socioemotional outcomes, the most common elements were homework tasks and log-keeping activities. Specifically, homework tasks were associated with interventions reporting poor efficacy for depression, anxiety, stress, and mindfulness, while self-monitoring activities were associated with interventions reporting poor efficacy for depression, anxiety, stress, wellbeing, and mindfulness (Grudin et al., 2022; Karyotaki et al., 2022; Küchler et al., 2023; Rodriguez et al., 2021; Sun et al., 2022). Log-keeping activities were associated with DMHIs reporting poor efficacy for depression, anxiety, stress, wellbeing, and mindfulness (Cook et al., 2019; Karyotaki et al., 2022; Klimczak et al., 2023; Koltz, 2022; Küchler et al., 2023; Schueller et al., 2019; Sit et al., 2022; Sun et al., 2022).

#### Elements Common to DMHI with Inconsistent Efficacy

Elements common to DMHI that yielded inconsistent socioemotional outcomes included psychoeducation, quizzes, and audio recordings. Psychoeducation was a common intervention element included in 13 studies (41.94%); however, psychoeducational contents and associated impacts on socioemotional outcomes were mixed. For instance, some studies found positive impacts on depression and anxiety levels (Juniar et al., 2022; Stapinski et al., 2021; Wahlund, 2022), while others reported negative impacts on depression and anxiety (Cook et al., 2019; Karyotaki et al., 2022; Pescatello et al., 2021). Similarly, interventions that contained quizzes reported positive impacts on depression and anxiety (Juniar et al., 2022; Stapinski et al., 2021), whereas others that contained quizzes demonstrated no evidence of efficacy on said outcomes (Karyotaki et al., 2022; Pescatello et al., 2021). Likewise, audio recordings were associated with both positive effects (Juniar et al., 2022; Sun et al., 2022; Wahlund, 2022) and non-significant effects on socioemotional outcomes (Küchler et al., 2023; Schueller et al., 2019).

## Discussion

This systematic review aimed to appraise the available literature on the socioemotional effectiveness of guided and partially guided digital mental health interventions (DMHIs) for indicated youth populations. Thirty-one studies from published and unpublished sources were identified that utilized guided or partially guided DMHIs for youth.

### Summary of Key Study Findings

A major and unique finding of this review was the identification of elements that were common to interventions demonstrating clinical efficacy and elements that were common to those demonstrating poor or yet to be established clinical efficacy. Within efficacious DMHIs for anxiety and depression, refresher/follow-up content, goal setting content, and relapse prevention content were common features. Additionally, content personalization and personalized recommendations were found in efficacious interventions for depression, stress, and wellbeing. Conversely, homework tasks, self-monitoring activities, and log-keeping activities were common to interventions reporting poor efficacy or yet to be established efficacy for the socioemotional outcomes of depression, anxiety, stress, wellbeing, and mindfulness. Across the socioemotional outcomes examined, the most common DMHI elements associated with the preservation of long-lasting impact were content personalization and self-reflective activities. This finding demonstrates the potential influence of intervention design decisions themselves on clinical outcomes. Further analysis of the impacts of intervention elements is warranted to inform developments in this field. This is the first study to attempt to draw associations between socioemotional outcomes and the specific DMHI elements. Given the clinical efficacy of personalized content and personalized recommendations, it can be highlighted that the digital health landscape has evolved from a ‘one-size-fits-all’ approach to more personalized care. As well, this systematic review is the first to examine the outcomes of stress, well-being, quality of life, and mindfulness in youth-specific guided DMHIs, with prior reviews examining a narrow range of socioemotional outcomes (depression, Välimäki et al., 2017; depression *and* anxiety, Ebert et al., 2015; Garrido et al., 2019).

### Comparing Results to Prior Research

We found that guided and partially guided DMHIs demonstrated consistent *short-term* improvements in several youth socioemotional outcomes, particularly for depression, anxiety, and stress. This is similar to prior systematic reviews in children and adolescents across various age groups (Grist et al., 2019; Hollis et al., 2017), and youth more specifically (Clarke et al., 2015; Välimäki et al., 2017), which assessed general DMHIs. When looking at *long-term* outcomes, DMHI efficacy inconsistencies were particularly prevalent for the outcomes of depression, anxiety, and stress, findings that have been reported in the existing youth-specific literature, with some indicating DMHI superiority relative to control groups (Clarke et al., 2015; Välimäki et al., 2017), while others showing no evidence of DMHI superiority at follow-up (Bennett et al., 2019; Grist et al., 2017). However, a limitation of the existing body of research included DMHIs primarily focused on the immediate program period, which did not provide ongoing support or assessment post intervention. In the present study, we observed heterogeneity in DMHIs, which may have contributed to observed inconsistencies, clouding true study findings. Factors contributing to these inconsistencies include content, intervention adherence, study design, content delivery method, and the presence of control groups. The dynamic and fast-evolving DMHI landscape has also contributed to this variability. However, as DMHI research amasses, a more granular systematic review of these programs will be able to take space, minimizing such variance.

Consistent with findings from adult reviews (Domhardt et al., 2019; Leung et al., 2022; Ma et al., 2021), we identified that the provider of guided DMHI human support (e.g., professional, peer, student), and their associated training or qualification level, did not appear to impact socioemotional outcomes. This appears to suggest the general value of human engagement and support within these digital interventions. These are promising findings as they may reduce the burden on mental health professionals while also offering less costly healthcare solutions.

Further, we identified that the delivery mode of human guidance, whether synchronous (e.g., videoconferencing) or asynchronous (e.g., text or email), did not appear to influence the mental health outcomes of depression, anxiety, stress, or well-being. This result aligns with adult-oriented reviews (Furness et al., 2020; Yellowlees et al., 2021). Drawing on asynchronous guidance has been associated with enhanced provider efficiency and participant flexibility (Lagera et al., 2023). However, it must be noted that we did observe a lack of entirely synchronous guided DMHIs in the present review (*k* = 4, 13%), a finding reported in a related review (Zhou et al., 2021). Despite limited data, synchronous youth DMHIs show promise in improving socioemotional outcomes, which is consistent with the broader youth-specific literature (Lattie et al., 2022; Li, 2023). Moreover, since a further four studies (13%) reported on entirely guided interventions, which offered no self-directed component, due to a lack of data we were unable to draw conclusions about their effectiveness compared to partially guided DMHIs that offered a combination of human support and self-guided program elements.

While we sought to examine *brief* interventions, no interventions with less than three sessions were identified, highlighting the under-explored potential of very brief or single-session interventions. This is important as adult research had identified that the modal number of therapy sessions attended is one, irrespective of client mental health diagnosis, severity, or complexity (Young et al., 2012). Further, research tells us that, on average, 75% of adults who ‘drop out’ from therapy after a single therapy session are happy with that one session (Barbara-May et al., 2018; Josling & Cait, 2018; Söderquist, 2018).

### Strengths and Limitations

A key strength of our review is its methodology, which includes a comprehensive search strategy, co-designed approach, diversity of included study designs, duplicate screening processes, appraisal of included studies, and inclusion of both published and unpublished literature from varied sources.

Despite strengths, limitations of the present review’s methodology must be noted. This systematic review was limited by its inclusion of internalizing socioemotional symptoms only, due to a lack of available data on externalizing symptom outcome data. Results are skewed by US-specific literature, which has notable cultural differences to other Anglophone countries including Australia. Finally, 48.39% of the review’s sample was drawn from university students. As the review examined youth ranging from 12-to-25 years, there are generalizability concerns for youth that are 12-to-18 years and 22-to-25 years who fall outside of the usual university enrolment years (Auerbach et al., 2016; Mortier et al., 2018). Due to a lack of identified data, we were unable to report on minority populations. Due to date restrictions, some pertinent studies may have been excluded that were published pre-2018. However, due to the substantial changes in the DMHI space in recent years, older studies are expected to have diminishing relevance.

### Recommendations for Improving Youth DMHIs

**Fig. 3:**
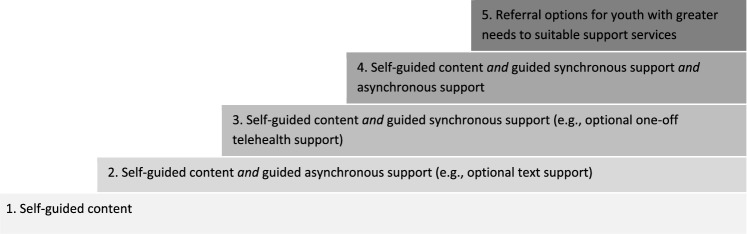
Tiered intensity model of digital support

**Fig. 4:**
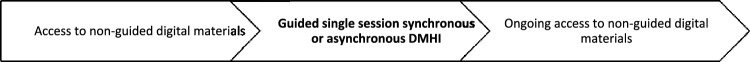
Bookending a guided single session DMHI with non-guided digital supports

In light of the review’s findings, recommendations are proposed:

1. *Integration of refresher and follow-up content* The short-term nature of the DMHIs’ socioemotional effects necessitates the incorporation of follow-up or refresher content. This could be in the form of periodic check-ins, booster sessions, or reminders that revisit key concepts.
2. *Re-engagement opportunities*
  - *‘The door is always open’* Embrace Single Session Thinking principles to convey that users can always return for support (Rycroft & Young, 2021). This approach reframes the concept of disengagement. Instead of viewing engagement lapses as failures, this perspective reframes disengagement positively, valuing each interaction as meaningful, regardless of frequency. It recognizes that users may have received the help they needed at that time, rather than seeing it as a ‘dropout’ or ‘failure to engage.’ This perspective encourages maximizing the benefit of each interaction, including digital ones, and reduces the stigma associated with re-engaging.
  - *Continuous access to content* Allow uninterrupted access to asynchronous DMHI content for users to re-engage at their convenience, acknowledging that mental health can fluctuate over time.
3. *Emphasis on goal setting and relapse prevention strategies* Interventions that include goal setting and relapse prevention content have shown efficacy. Goal setting helps individuals stay focused and motivated, while relapse prevention strategies can aid in maintaining gains over time.
4. *Re-evaluation of homework and monitoring elements* Given the suboptimal efficacy correlated with certain DMHI elements including homework tasks, self-monitoring, and log-keeping activities, a reassessment and potential reconfiguration or reduction of these DMHI elements is warranted. Ensuring these elements are not overly burdensome and are clearly linked to therapeutic goals is necessary.
5. *Enhancement of user engagement strategies* To counteract the identified fleeting nature of DMHI efficacy, innovative strategies to bolster user engagement are imperative. This may encompass interactive features, gamification elements, or personalized content.
6. *Continuous evaluation and refinement of active components *via* longitudinal studies* To better understand the long-term effects of these interventions, longitudinal studies are needed. This can help in identifying which components have lasting impacts on socioemotional outcomes.
7. *Focus on accessibility and user-friendliness* Ensuring that DMHIs are accessible and user-friendly appears crucial in reducing attrition rates and enhancing overall socioemotional effectiveness.
8. *Optimizing DMHI delivery and intervention strategies*
  - Given the mode of DMHI delivery (i.e., asynchronous, synchronous, combined) did not appear to notably impact socioemotional outcomes, focus on developing and implementing more novel, flexible, and cost-effective delivery methods. This approach should aim to maximize accessibility and convenience for users, while also considering the operational efficiencies for providers. For example, place greater emphasis through those with a lived experience, rather than reliance on therapists.
  - Since the type of support personnel (e.g., therapist, researcher, peer, student) did not appear to influence outcomes for this population, concentrate on optimizing the duration and intensity of DMHIs for a balance between effectiveness and user engagement.
9. *Pre, post, follow-up DMHI evaluations* To understand and address user outcomes, it is essential to gather data both before and after they participate in the DMHI. This pre- and post -intervention data collection will likely provide valuable insights into user requirements, helping to tailor the DMHI more effectively to meet these needs. It is crucial to employ validated and reliable instruments for assessing client progress and reflection, not only during the intervention period but also for an extended duration of 1–2 years post intervention. It is further necessary to understand the effectiveness of a DMHI over an extended period of time, to learn when re-engagement might be indicated as effects wear off, for example.
10. *Content personalization and being client-led* DMHI content personalization involves designing and adapting the intervention content to align with the individual needs, preferences, and circumstances of each user. Personalized content can be achieved through initial evaluations of the user’s specific mental health challenges, preferences in learning and engagement, and unique life circumstances. This approach is expected to increase user engagement, satisfaction, and overall effectiveness of the intervention.
11. *Developmentally suitable* Youth and young adulthood encompasses a wide age range, necessitating the consideration of developmentally appropriate DMHI content through a life course developmental lens.
12. *Incorporation of feedback mechanisms* Embedding automated and human-led feedback channels to listen to the client creates a client-informed service may enhance their socioemotional efficacy. These systems, which can include options for anonymity, serve to both continuously improve the intervention and tailor it to individual user needs.
13. *Mobile app-based content* As digital interventions evolve from web-based to app-based formats, incorporating mobile app content in new DMHIs for youth becomes crucial. This aligns with young users’ expectations and boosts engagement. App-based platforms offer flexibility in synchronous and asynchronous support, catering to individual needs and schedules. They also provide opportunities for interactive features and gamification to further engage users.
14. *Leveraging smartphone capabilities* Smartphones’ built-in features offer valuable opportunities for improving DMHIs. Examples include:
  - *Location services for resource connectivity* Leverage the smartphone’s location capabilities to connect users with local mental health services, youth facilities, and safe social venues, facilitating easy access to nearby support and resources.
  - *Gamification through token economy* Integrate a token economy (Kazdin, 1977) within apps to make progress tracking more engaging. For example, youth can earn tokens for each day they avoid behaviors (e.g., self-harm, binge-purging). This could be paired with easy re-engagement options and the normalization of re-engagement with a service.
  - *Movement tracking to promote healthier lifestyles* Use phone’s movement monitoring capabilities to motivate users to increase their physical activity, which has been associated with mental health improvements in youth (Rodríguez-Romo et al., 2022).
15. *Gaps in research literature and existing brief guided DMHIs*
  - *Trauma-informed DMHIs* No study reported explicitly on trauma-informed elements, the critical importance of this orientation is now undisputed in mental health intervention literature (Sockolow et al., 2017; Ting & McLachlan, 2023). Thus, there is a need for research to align with clinical insights more closely and overtly on trauma-informed practices, as well-documented in victimisation, trauma, and long-term treatment literature. As an example, in a trauma-informed care approach for single session encounters one key consideration could be to avoid requiring clients to repeatedly recount their mental health history if they happen to engage with multiple different practitioners. This is because such a requirement can potentially be retraumatising (Frueh et al., 2005).
  - *Co-designed DMHIs* should be explored in more depth. This involves incorporating feedback from current and former clients, practitioners, and client support systems when developing and revising DMHIs. This process should also consider cultural safety by including diverse cultural and population consultations.
  - *Peer support and engagement* Research has yet to fully explore the benefits of peer support and engagement, especially as an initial engagement strategy before clinical contact. Potential benefits include normalising problems, reducing hierarchical dynamics, cost-effectiveness, and improving accessibility. Within an intervention, this could include considering online communities within a DMHI (e.g., live online chat group, asynchronous moderated discussion forums) to assist engagement and positive outcomes that may also provide a mechanism for long-term support without adding to the burden on clinical teams*.*
16. *Emphasising a strengths-based approach* This involves reminding clients of their personal resources and capabilities. Innovative methods such as automated games or digital interactive activities can be utilised to reinforce the client’s sense of self-efficacy and remind them that they possess the solutions to many of their challenges. This approach aims to boost client confidence and promote a self-reliant perspective in addressing their issues.
17. *Implementing a multi-tiered support model* We recommend a multi-tiered DMHI support model, allowing for tailored intervention and intensity based on user needs, utilizing the full spectrum of the digital ecosystem. Figure 3 illustrates a multi-tiered model of care wherein graded referral or progression pathways are made based on need. This approach would conserve professional and financial resources for those most in need. Client’s may complete a pre-DMHI questionnaire to inform the optimal pathway through the tiered structure, as well as additional check-ins to monitor for the need to increase or reduce support.
18. *Bookending guided digital support with complimentary non-guided digital resourses* Bookending existing guided (a/synchronous) Single Session approaches with access to non-guided digital online resources could be beneficial. Following a trauma-inform stance, digital non-guided resources could be client-selected. Figure 4 displays a basic example of this approach.
19. *Systemic awareness and responsiveness* Service providers have systems in place for effective and coordinated communication that facilitates the delivery of safe and high-quality care for service users and *their support network*. This will allow for the provision of wraparound support not placing all the responsibility on the vulnerable young person.
20. *When additional support is required*
  - *Brief DMHIs as a gateway into longer-term support* Brief digital work can serve as an initial step, providing a gateway to longer-term treatment options or facilitating referrals to other appropriate services. This role positions brief interventions as a critical entry point in a broader therapeutic process.
  - *Referrals* Incorporating high-quality referral sources and systems. This approach ensures that clients are directed to the most appropriate resources or services, fostering a comprehensive care strategy that extends beyond the brief DMHI.

### Future Research

Future research will be strengthened and refined through the inclusion of externalizing socioemotional outcomes, permitting a more robust analysis of youth socioemotional outcomes. Future research may also consider exploring DMHI user experience elements (e.g., intervention feasibility, satisfaction, retention, engagement, credibility, motivation). These user experience efficacy outcomes are as critical as the socioemotional outcomes examined in the present review and are two sides to the same coin: both outcomes must balance in harmony for these programs to work successfully. Further research is also required to assess the utility of current DMHIs for diverse populations, including culturally and linguistically diverse communities, diverse socioeconomic groups, and those based in rural or regional locations. Further, modifications of existing interventions or the formation of specific DMHIs for diverse populations is required to enhance factors such as engagement, use, relevance, and trust. Once developed, these will require assessments of efficacy. Further, as we did not identify a brief intervention with less than three sessions, this highlights the under-explored potential of single session or very brief digital mental health interventions for youth that are evidence-based and grounded in science, a notable gap in the literature. Finally, more research on long-term follow-up (i.e., up to 12 months post intervention) is needed to track the enduring or decaying nature of intervention effects.

Results highlight important practice implications, including the value of program engagement with youth using these types of interventions, the need for individualized DMHI content for youth, and the need for ongoing follow-up or refresher program content to ensure sustained intervention effects. While findings were generally similar to other reviews (Clarke et al., 2015; Välimäki et al., 2017), as the needs and context of youth are often unique, this study offers a developmentally specific account of youth DMHIs. This review provides important implications for future investment to design a new digital health model of care for youth that combines both refresher/follow-up content, goal setting, and relapse prevention content together with content personalization and personalized recommendations. Combining the successful elements of DMHIs has the potential to lead to useful interventions for this population.

Study findings are being utilized by our key stakeholder, Beyond Blue, to inform the continuous improvement of their Community Support Services model of care, including how it can meet the needs of younger people. Beyond Blue offer single session, brief interventions using phone, email, and webchat provided by an accredited counsellor workforce and trained coaches.

## Conclusions

The finding from this systematic review serves as a promising evidence-base from which further empirical studies can be conducted. While some results were varied, there was strong evidence that these programs are effective for depression, stress, and anxiety outcomes, but that these were short-lived. We also provide an initial examination of the specific DMHI elements common to interventions that yielded positive or negative socioemotional intervention outcomes. This represents an important move toward strengthening evidence-enriched digitally focused mental health services for youth and young adults. Further quality research is necessary before we can determine the socioemotional outcomes associated with DMHIs.

### Supplementary Information

Below is the link to the electronic supplementary material.

Supplementary file1 (DOCX 39 KB)
